# High Diversity and Low Genetic Differentiation Among Geographic Populations of *Myotis yumanensis* in Western Canada

**DOI:** 10.3390/ani15040578

**Published:** 2025-02-18

**Authors:** Xingyuan Su, Nicolas Popescu, Chadabhorn Insuk, Cori Lausen, Jianping Xu

**Affiliations:** 1 Department of Biology, McMaster University, Hamilton, ON L8S 4K1, Canada; sux21@mcmaster.ca (X.S.); popescun@mcmaster.ca (N.P.); insukc@mcmaster.ca (C.I.); 2 Wildlife Conservation Society Canada, P.O. Box 606, Kaslo, BC V0G 1M0, Canada; clausen@wcs.org

**Keywords:** Yuma Myotis, microsatellite loci, genetic diversity, population structure, white-nose syndrome

## Abstract

*Myotis yumanensis* is a small insectivorous bat distributed in Western North America and is susceptible to white-nose syndrome, a devastating fungal disease. Effective management of the disease in *M. yumanensis* requires an understanding of its population structure to identify possible routes and barriers of disease transmission. Here we used nine microsatellite loci to investigate genetic variation among 336 *M. yumanensis* from 10 locations in Western Canada. Our analyses revealed high genetic diversity and low but statistically significant genetic differentiation among several geographic populations. Interestingly, though geographically it was not the most distant, the Lillooet population showed the highest genetic differentiation from others, suggesting factors other than geographic distance also contributed to gene flow in this species. While white-nose syndrome has been reported in *M. yumanensis* populations in neighbouring Washington State in the United States of America, as of 2023, the causative pathogen was not detected on the wings of our analyzed 336 *M. yumanensis* bats in Western Canada. However, continuing monitoring is required to provide the most updated information about the white-nose syndrome status in these populations. The knowledge of *M. yumanensis* population structure in Western Canada will help develop effective management strategies for protecting this bat species from white-nose syndrome.

## Simple Summary

Bats are keystone species and play important roles in natural ecosystems. There are at least 18 bat species in Canada, and the highest species diversity is found in British Columbia in Western Canada. At present, bats in 9 of the 10 Canadian provinces (except British Columbia) and 40 of the 48 continuous continental states in the United States of America have been reported to suffer from white-nose syndrome, a deadly infection caused by the fungus *Pseudogymnoascus destructans*. Yuma Myotis (*Myotis yumanensis)* is a common bat species in Western Canada that contributes significantly to insect pest control but is susceptible to white-nose syndrome. However, there is so far very limited genetic knowledge about this bat, including its population structure, especially in Western Canada. By analyzing 336 *M. yumanensis* from 10 geographic locations in Western Canada using nine nuclear microsatellite loci, this study revealed high genetic diversity within most geographic populations and low genetic differentiation among geographic populations. Specifically, differences in the levels of genetic differentiation between interior and coastal populations were observed. Our results on population structure and potential gene flow among different areas will be helpful for the management of white-nose syndrome.

## 1. Introduction

There is increasing recognition that biodiversity is important to ecosystem integrity and human health [1]. Wildlife diseases continue to increase, threatening fauna around the globe [2]. Understanding the pattern of disease transmission through host movement patterns is essential for population conservation and management before and during disease outbreaks. Genetic methods offer a helpful alternative to traditional demographic approaches for examining host movements and their impact on disease transmission. This is based on the assumption that rates of contact among individuals resulting in gene flow are indicative of contacts that could lead to disease transmission [3,4]. Such has been the case with bats and the most catastrophic disease in North America—white-nose syndrome (WNS)—where bat-to-bat contact has been implicated in the rapid spread of the fungal disease agent *Pseudogymnoascus destructans* [5].

Bats (order Chiroptera) are a diverse and large group of mammals important for bioresources worldwide, such as food crops and medicinal plants, through their provisioning of ecosystem services, including natural pest control and pollination [6,7,8]. In the United States of America, increased pesticide use in response to reduced bat populations has been implicated in higher human infant mortality rates [1], emphasizing the important One Health connection between bat populations and humans. Because of bats’ nocturnal lifestyle and cryptic behavior, it is often difficult to track their movements both spatially and temporally. Evidence from studies employing population and landscape genetics has demonstrated that landscape features, such as mountains, can limit host gene flow and thus potentially act as barriers to disease transmission [4,9]. In areas where WNS has not yet been detected, understanding gene flow in lesser-studied bat species can shed light on potential disease spread and proactive management strategies.

Yuma Myotis (*Myotis yumanensis* H. Allen, 1864) is a small bat with a geographic distribution restricted to the western part of North America, from Alaska to Mexico [10,11]. It is similar in morphology to the trans-continental *M. lucifugus*, which causes difficulty in distinguishing these two species in the field [11,12]. In addition, these two species frequently roost together in the same maternity roosts [13]. In North America, diagnostic symptoms of, and mortality from, WNS have been confirmed in both of these bat species [14,15]. Although *M. yumanensis* is currently categorized as “Least Concern” by the International Union for Conservation of Nature [16], this species faces looming threats of WNS, habitat loss, and climate change, and some state legislatures have thus listed the species as “Special Concern” [15]. Typically found co-roosting with *M. yumanensis*, *M. lucifugus* is “Endangered” under the federal *Species at Risk Act* in Canada (since 2014) due to dramatic declines in populations across its eastern range, caused by WNS. As rates of WNS-caused mortalities of both species have been similar in Washington state of the United States of America, the only western location where WNS mortalities have been reported to date for *M. yumanensis* (A. Tobin, Washington Fish and Wildlife, pers. comm.), the disease outcome is predicted to be similar for these two species. While genetic studies predicting disease spread have been conducted for *M. lucifugus* [3,5,9,17], no such studies have been done for *M. yumanensis*.

Although *M. yumanensis* is a widely distributed species, few individuals have been genetically studied. The whole genome sequence of the first *M. yumanensis* was recently obtained and assembled for a juvenile male bat captured in Chester, Plumas County, California [18]. However, most genetic studies of *M. yumanensis* have focused on taxonomy and systematics within this species and between *M. yumanensis* and other closely related species [11,19]. Interestingly, a large-scale phylogenetic assessment of the *Myotis* genus in 2024, including relatively few individuals from each species, revealed many outstanding issues with regard to the delimitations and relationships among species within this genus, underscoring the critical importance of population genetics studies to determine the geographic distribution range and population structure and reproductive behavior of each species in nature [19]. Given the importance of ecological services provided by bats, extinction risks posed by WNS on susceptible bat species, and poor knowledge of the population genetics of *M. yumanensis*, it is urgent to evaluate the genetic diversity and population structure of *M. yumanensis* before WNS becomes widespread across Western North America. To initiate this process, we investigate the genetic population structure of *M. yumanensis* based on bats captured between 2022 and 2023 in British Columbia, the only province in Canada that has not yet confirmed the presence of WNS [20].

As this may be the first population genetics study of this species, and because wing sampling included tissue biopsies and wing swabs, we asked the following four questions: (1) Can multilocus microsatellite markers be used to separate *M. lucifugus* and *M. yumanensis*, a necessary step before data analysis due to potential species identification error caused by morphological similarity between the two species [11,12]? To address this question, we used the microsatellite markers originally developed for *M. lucifugus* to genotype samples of both *M. lucifugus* and *M. yumanensis* and analyze their genetic clustering pattern. (2) Is the Western Canadian population of *M. yumanensis* a randomly mating population? To address this question, we conducted two tests: the Hardy–Weinberg equilibrium test and the genotypic linkage disequilibrium test. In the Hardy–Weinberg equilibrium test, we investigated whether the observed associations of alleles at each individual locus deviated from the expected genotype frequencies generated based on the null hypothesis of random mating. In the genotypic linkage disequilibrium test, we compared the observed genotype associations between pairs of loci with the expected frequencies generated based on random recombination and independent assortment. (3) How does geographic separation contribute to genetic variation in the total Western Canadian *M. yumanensis* population? To address this question, we conducted multiple population genetics tests, including STRUCTURE clustering, analysis of molecular variance (AMOVA), pairwise population differentiations (*F*_ST_), and the Mantel test between geographic distance and genetic distance. We hypothesize that the western *M. yumanensis* populations are panmictic and will exhibit high genetic diversity and evidence for gene flow among geographic regions despite extensive mountainous terrain, due to the high mobility of bats [21]. (4) Is the fungal pathogen *P. destructans* present on the wings of *M. yumanensis* in Western Canada? Because WNS has been found in *M. yumanensis* in the adjacent Washington State of the United States of America, we hypothesized that this fungal pathogen might be present on the wings of some *M. yumanensis* in British Columbia.

## 2. Materials and Methods

### 2.1. Collection of Wing Swabs and Wing Biopsy Punches from Bats

Bats were caught from April to September in 2022 and from April to August in 2023 in southern British Columbia, Western Canada, by researchers and volunteers associated with the Wildlife Conservation Society Canada. For each bat, wing swabs were collected and stored following methods described in Insuk et al. [22]. Each wing swab was placed into a sterile Eppendorf tube and stored in a −20 °C freezer within 2–8 hours after sampling. Two wing biopsy punches (2 mm in diameter) were collected from each captured bat and stored in 100% ethanol in a 1.5 mL sterile microcentrifuge tube. Bats were presumptively identified to species based on their morphological and echolocation features as illustrated in the book “Bats of British Columbia” [21]. However, as misidentification of *M. yumanensis* and *M. lucifugus* can occur due to overlapping morphological and echolocation traits [23], all captured bats tentatively identified to these two species were included in our initial genetic screening. In the field, bats tentatively identified as *M. yumanensis* were labelled as “MYYU”; those tentatively identified as *M. lucifugus* were labelled as “MYLU”. When a bat could not be distinguished between *M. yumanensis* and *M. lucifugus*, the sample was labeled as “MYYU/MYLU” or “unidentified”.

A total of 721 bats labelled with “MYYU”, “MYLU”, “MYYU/MYLU”, or ”unidentified” were included in our initial genetic screening (please see details below in Section 2.4). However, only MYYU samples identified based on both morphological features and genetic features would be included for further population structure analyses (for details, please see Section 2.4 below). Bat capture and sampling was conducted under permit number MRCB20-598305 (issued by the Ministry of Forests, Lands, and Natural Resource Operations of the Province of British Columbia to Wildlife Conservation Society Canada, permit approved on 27 March 2020, permit effective between 1 April 2020 and 31 March 2025). To help protect the bat colonies from unwanted attention and to protect the privacy of landowners where some of the bat colonies resided, the exact latitude and longitude information of our sampling locations will not be released.

### 2.2. DNA Extraction

We extracted DNA from one wing biopsy punch using the QIAGEN DNeasy Blood & Tissue Kit (Cat. No. 69504 and 69506) (QIAGEN, Hilden, Germany), which used a silica-based process in spin-column format. We followed the DNeasy Blood & Tissue Kit Quick-Start Protocol as described on the QIAGEN website for all samples. In addition, we also used spin columns from Omega Bio-Tek Blood DNA Extraction Kits (Omega Bio-Tek, Norcross, GA, USA) from Avantor Company (Catalog # CA95043-212) with QIAGEN reagents for DNA extractions.

### 2.3. Microsatellite Genotyping

Through literature search, we found 11 nuclear tetranucleotide microsatellite loci characterized for *M. lucifugus* by Burns et al. [24]. The final set of microsatellite loci used for the population genetics of *M. yumanensis* was determined based on the amplification and genotyping success of the microsatellite loci and whether the locus showed polymorphism among individuals (See Results section for which loci were retained). We optimized multiplex polymerase chain reactions (PCRs) based on published multiplex information of these 11 loci [25] and the multiplex PCR protocol [26] to analyze all 721 bats. Within each optimized multiplex, a unique fluorescent dye was assigned to each primer pair to label each locus with a different color. Multiplex PCR products were assessed through agarose gel electrophoresis and diluted before they were sent to Mobix Lab at McMaster Genomics Facility (McMaster University, Hamilton, Canada) for fragment analysis. Mobix Lab used Applied Biosystems Genescan 600 LIZ Size Standard (catalog number: 4366589) or Applied Biosystems GeneScan 600 LIZ Dye Size Standard v2.0 (catalog number: 4408399) (Thermo Fisher Scientific, Waltham, MA, USA). For each sample, 9 μL of size standard mixture (8.75 μL highly deionized (Hi-Di) formamide and 0.25 μL LIZ 600 size standard) was combined with 1 μL PCR-amplified DNA sample in a well on a microtiter plate, which was heated in a thermocycler (96 °C for 3 min to denature the DNA double strands) and then cooled for 2 min on ice. DNA fragments were separated based on their sizes using capillary electrophoresis (3730 Genetic Analyzer, Applied Biosystems POP-7 Polymer, catalog number: 4363929; Thermo Fisher Scientific, Waltham, MA, USA). We analyzed output files using Open Source Independent Review and Interpretation System (OSIRIS) software (Version 2.16) using LaneStandardOnly 5 Operating Procedure and ABI-LIZ-600-60-TO-600 Internal Lane Standard [27,28]. We manually recorded raw sizes (with decimals) of probable alleles and performed allele binning (round to integer) first by TANDEM software (Version 1.09) [29], followed by manual binning based on the TANDEM’s results: we accounted for single base pair (bp) artifacts off of the true allelic peak [27], by binning allele sizes differing by at most 1 bp as the same allele, with potential further adjustments if the size range was large. Although this binning method could underestimate the number of alleles and thus the amount of genetic variation, this method was conservative, contributing to more robust conclusions.

### 2.4. Genetic Screening: Exclusion of Non-M. yumanensis Samples

Because *M. yumanensis* and *M. lucifugus* are morphologically similar and can be difficult to distinguish in the field, we first tried to filter potential misidentifications and remove them from the final *M. yumanensis* dataset, a necessary step to ensure no potential inclusion of two species in the analyses that could overestimate genetic variation and lead to wrong conclusions about population structure. We used the software STRUCTURE (Version 2.3.4) [30] for this task, which assigned individuals to possible genetic clusters based on genotypes using the Bayesian clustering method. By using STRUCTURE, we predicted *M. yumanensis* and *M. lucifugus* to separate into two distinct genetic clusters. A total of 721 bats of both *M. yumanensis* and *M. lucifugus* were included in the screening step because we found that including morphologically identified *M. lucifugus* would help the clustering and thus the removal of non-*M. yumanensis* samples. We selected an admixture model that assumed individuals to have mixed ancestry, allowing us to identify and exclude potential hybrids between the two species. We also used the correlated allele frequencies model [31], which assumed similar allele frequencies in different genetic populations. The number of genetic populations (*K*) selected for STRUCTURE to analyze was *K* = 1 to *K* = 10, and STRUCTURE was run 15 times for each *K*. Our burn-in length was 10^5^ with an additional 10^6^ cycles to allow the analyses to reach a stable optimum *K* value.

### 2.5. Allelic Diversities and Genotyping Error Estimation

After excluding potentially misidentified *M. yumanensis,* we performed the following allelic diversity and genotyping error analyses. For quantitative estimates of allelic diversity at these microsatellite loci, we used the poppr (Version 2.9.6) [32,33] and polysat (Version 1.7.7) packages [34,35] in R (Version 4.4.1) [36]. At each locus, we calculated the number of alleles, observed heterozygosity, expected heterozygosity, and polymorphism information content (PIC). To quantify genotyping errors, we calculated the mean error rate per allele and the mean error rate per locus following the method described by Pompanon et al. [37].

### 2.6. Tests of Hardy–Weinberg Equilibrium and of Linkage Disequilibrium

To investigate whether the total population and individual geographic populations were randomly mating, we tested whether the observed genotype frequencies deviated from Hardy–Weinberg equilibrium at each locus and whether there were significant heterozygote deficiency or heterozygote excess in the total sample as well as in each geographic population, respectively (See Table 1 and the Results section for the definition of the geographic populations). The Hardy–Weinberg equilibrium test and the exact *p*-values were performed and estimated by the Markov chain method in the Genepop program (Version 4.8.3) [38]. Markov chain parameters for all three tests (Hardy–Weinberg equilibrium, heterozygote deficiency, heterozygote excess) were 10,000 dememorizations, 100 batches, and 5000 iterations per batch. Correction of *p*-values for multiple comparisons was done using the p.adjust function with the Benjamini and Hochberg method in the stats R package (Version 4.4.1) [36]. Corrected *p*-values below 0.05 were used to reject the null hypothesis of Hardy–Weinberg equilibrium. Within-population fixation index *F*_IS_ estimates were also computed using Weir and Cockerham’s estimator [39] and Robertson and Hill’s estimator [40] by Genepop (Version 4.8.3).

We further tested whether genotype frequencies between pairs of microsatellite loci deviated significantly from random association. This test was performed for the total population as well as for each geographic population separately (See Table 1 and the Results section for the definition of the geographic populations), with statistical significance derived using the Markov chain method in Genepop (Version 4.8.3). Markov chain parameters were 10,000 dememorizations, 100 batches, and 5000 iterations per batch. Correction of *p*-values for multiple comparisons was done using the p.adjust function with the Benjamini and Hochberg method in the stats R package (Version 4.4.1) [36]. Corrected *p*-values below 0.05 were used to reject the null hypothesis of independence of genotypes between pairs of loci.

### 2.7. Estimating Population Structure in M. yumanensis

To investigate whether the total sampled individuals from Western Canada belonged to one or more genetic populations, we applied the STRUCTURE clustering method and analysis of molecular variance (AMOVA) to estimate the population structure in the total *M. yumanensis* samples. STRUCTURE software was used to find the optimal number of genetic populations in our samples of *M. yumanensis*. The parameters of STRUCTURE were the same as in Section 2.4. We also ran STRUCTURE using the LOCPRIOR models, with geographic populations (See Table 1 and the Results section for the definition of the geographic populations) as prior information to improve clustering of individuals in the situation of weak population structure [41]. We performed AMOVA using the poppr.amova function in the poppr R package (Version 2.9.6) [32,33], with clone-correct set to TRUE, the method of correcting missing dataset to “genotype”, and variance within individuals set to FALSE, and the AMOVA method set to “pegas”.

We further used pairwise *F*_ST_ to estimate population differentiation between pairs of geographic populations (See Table 1 and the Results section for the definition of the geographic populations). We calculated pairwise *F*_ST_ (Weir and Cockerham‘s method, which accounted for sample sizes [39]) using the pairwise.WCfst function in the hierfstat R package (Version 0.5.11) [42]. Statistical significance of the estimated *F*_ST_ values was determined using 1000 bootstraps over loci using the boot.ppfst function in the hierfstat R package (Version 0.5.11) [42].

### 2.8. Test of Isolation-by-Distance and Isolation-by-Landscape Connectivity Models

To investigate how geographic factors might have contributed to the patterns of genetic variation, we tested the isolation-by-distance model and the isolation-by-landscape connectivity model. More specifically, for the isolation-by-landscape connectivity model, since *M. yumanensis* is mostly found at lower elevations [11] while most of British Columbia is mountainous, we investigated the effect of elevation on the spatial pattern of genetic differentiation.

For testing the isolation-by-distance model, we calculated the geographic distance matrix consisting of great-circle distances between pairs of geographic populations using the pointDistance function in the raster R package (Version 3.6.26) [43], with longlat set to TRUE. We calculated the genetic differentiation (*F*_ST_) matrix using pairwise.WCfst function in the hierfstat R package (Version 0.5.11) [42].

For testing the isolation-by-landscape connectivity model, we calculated the geographic distance matrix consisting of least-cost distances based on elevation between pairs of geographic populations. We obtained the Canadian Digital Elevation Model as a raster layer of elevation using the cded_terra function in the bcmaps R package (Version 2.2.0) [44]. We converted negative values in the raster data to 0 using the clamp function in the terra R package (Version 1.7.78) [45]. To decrease the computational time, we aggregate raster data and reduce the number of cells about 100 times (914,433,310 cells to 9,149,193 cells) using the aggregate function in the terra R package, with fact set to 10 (Version 1.7.78) [45]. We created a transition (conductance) matrix by treating elevation values as resistance values (conductance values in the transition matrix were calculated as 1/resistance) using the transition function in the gdistance R package (Version 1.6.4) [46], with the “TransitionFunction” parameter set to “function(x) 1/mean(x)” and “directions” set to 8. We corrected the transition matrix for distance distortion using the geoCorrection function in the gdistance R package (Version 1.6.4) [46], with “type” set to “c” and “scl” set to TRUE. We calculated the least-cost distances between each pair of geographic populations using the costDistance function in the gdistance R package (Version 1.6.4) [46]. The genetic differentiation (*F*_ST_) matrix was calculated using the pairwise.WCfst function in the hierfstat R package (Version 0.5.11) [42].

We then applied the Mantel test to investigate the relationship between the two types of geographic distance matrices (great-circle distance and least-cost distance) and the pairwise *F*_ST_ matrix, respectively, using the mantel function in the vegan R package (Version 2.6.8) [47], with 999 permutations and Spearman’s correlation. We rejected the null hypothesis of no relationship in the Mantel test if the *p*-value was less than 0.05.

### 2.9. Real-Time-Quantitative PCR (qPCR) Procedure for Detection of P. destructans

To investigate whether the fungal pathogen *P. destructans* was present in any sampled individual bats, we performed qPCR testing using species-specific primers and probes to detect the presence of *P. destructans*. We used the following procedure for this test: for each wing swab (30 cm^2^) of each bat, which was stored in a 1.5 mL sterile tube, we added 450 μL Tris-EDTA (TE) buffer to suspend the swab, then took an aliquot of 6 μL as qPCR template. The full method was described in Insuk et al. [22]. Briefly, we used the conidia of the *P. destructans* strain US-15 at 10^4^, 10^5^, 10^6^, and 10^7^ spores/mL as positive controls and UltraPure water (Invitrogen) as a negative control. qPCR was performed in triplicates, and standard practice was performed to prevent contamination. qPCR was run in a CFX Opus 96 qPCR machine (Bio-Rad) using the SYBR/FAM channel. Results were analyzed in Bio-Rad CFX Maestro software (Version 2.3 standard edition) (Mississauga, ON, Canada). We considered any reaction that crossed the threshold baseline within 40 cycles to be positive. From the three replicates, we considered the swabs were positive for *P. destructans* if at least 2 out of 3 swabs showed a critical threshold (Ct) cycle of <40 in *P. destructans* detection.

## 3. Results

### 3.1. Optimizing Microsatellite PCR Conditions

Three multiplex PCRs were optimized to amplify 11 microsatellite markers. Volumes of reagents for optimized multiplex PCRs are listed in Table A1. Primer sequences and corresponding fluorescent dyes were listed in Table A2. Three multiplexes used the same PCR program in the SimpliAmp Thermal Cycler (Thermo Fisher Scientific, Waltham, MA, USA) to genotype all 721 bats (Table A2). The success of PCR was checked by agarose gel electrophoresis to determine dilutions of each sample for optimal fragment analyses. The diluted PCR products were sent for fragment analysis. Multiplexed PCR products in each mix showed relatively similar intensity of bands on the agarose gel, which indicated successful multiplexed reactions at similar efficiency. However, Mluc21 was poorly amplified in many samples and was excluded, leaving data on 10 microsatellite loci for the analyses described in Section 3.2.

### 3.2. Genetic Screening: Exclusion of Non-M. yumanensis Samples

**Figure 1:**
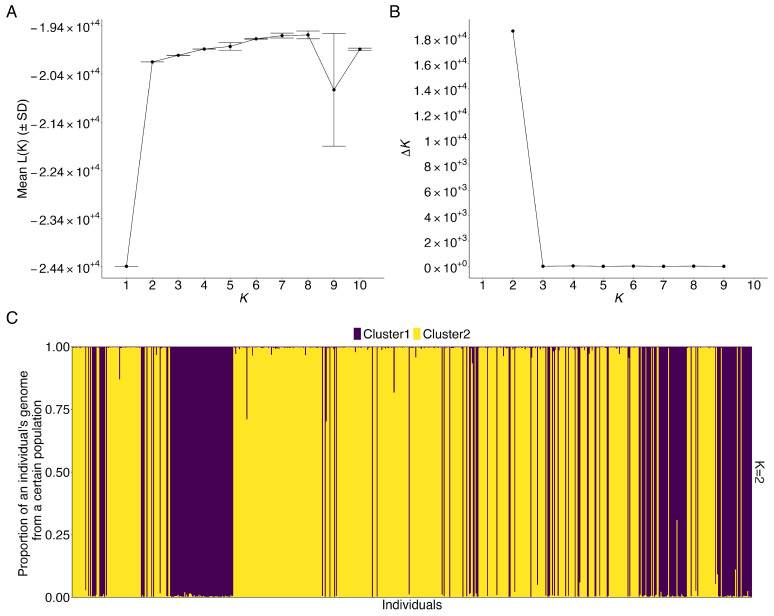
STRUCTURE results of 721 bats of *M. yumanensis* and *M. lucifugus*. (**A**) Mean log likelihood over 15 replicate runs at each user-defined number of genetic populations (K). Error bar represents standard deviation; (**B**) Second order rate of change of likelihood (∆K) defined by Evanno et al.; (**C**) Merged barplot from 15 barplots from 15 replicate runs showing individual assignment to 2 genetic clusters.

The initial STRUCTURE analysis was performed for all 721 bats belonging to *M. yumanensis* and/or *M. lucifugus* using genotype data at 10 microsatellite loci. Output files from STRUCTURE were read and analyzed using the pophelper R package (Version 2.3.1) [48]. Based on a method by Evanno et al. [49], the delta*K* value was maximum at *K* = 2 (Figure 1B), indicative of two distinct genetic populations (in this case, two species). Fifteen bar plots from 15 replicate runs were merged into one bar plot using the mergeQ function (Figure 1C), and this graph showed that most of the individuals were assigned to one of the two genetic clusters, corresponding to the two species.

**Figure A1:**
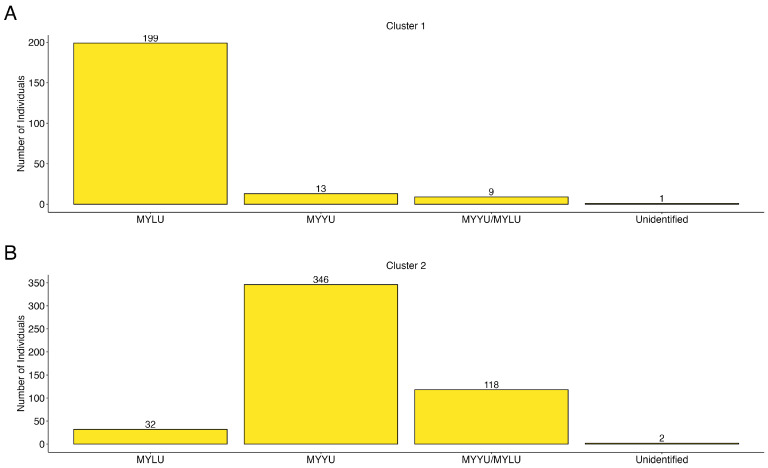
Number of different species identified by researchers in the field in each of the 2 genetic clusters, the best number of genetic populations identified by STRUCTURE software. (**A**) Number of different species in cluster 1; (**B**) Number of different species in cluster 2. MYYU = bats tentatively identified as *M. yumanensis*; MYLU = bats tentatively identified as *M. lucifugus*; MYYU/MYLU, Unidentified = bats that could not be distinguished between *M. yumanensis* and *M. lucifugus*.

Specifically, using 70% as the threshold of the proportion of an individual’s genotype to assign an individual to one of the two populations, we found cluster 1 contained a total of 222 individuals, cluster 2 contained a total of 498 individuals, and one individual was assigned to neither cluster (a putative admixed individual), which may represent a hybrid or a different Myotis species incorrectly identified in the field. In cluster 1, 199 out of 222 individuals were identified as *M. lucifugus* based on morphological and echolocation features as determined in the field (Figure A1). In cluster 2, 346 out of 498 individuals were identified as *M. yumanensis* based on field observations (Figure A1). Therefore, the results indicated that these two species were mostly separated into two clusters. Some specimens clustered differently than their field identifications. The true identities of these specimens require further genetic and genomic analyses. For subsequent population genetics analyses of *M. yumanensis*, only 346 *M. yumanensis* that showed consistent identification based on both field observations and STRUCTURE results were kept for subsequent analyses. The remaining data will be combined with other data and be analyzed in a separate study.

### 3.3. Sampling Sites and Geographic Populations of M. yumanensis

**Figure 2:**
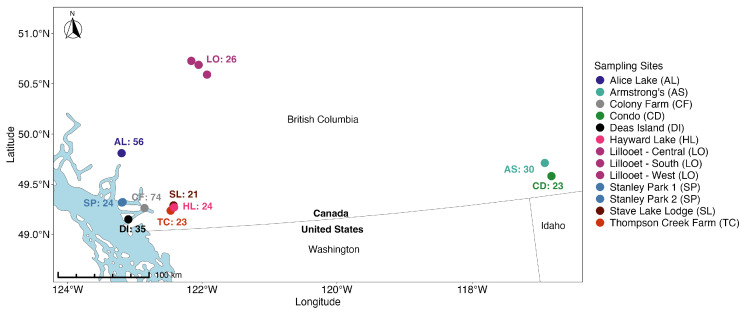
Map locations of 10 geographic populations of *M. yumanensis* and corresponding sampling sites in British Columbia, Canada. Sample size was labeled on the map. The map was produced using bcmaps R package (Version 2.2.0) [44] and sf R package (Version 1.0.17) [50,51] using the latitude and longitude of the sampling sites and the World Geodetic System 1984 (WGS84) coordinate reference system.

Among the 346 *M. yumanensis* bats, we excluded 10 individuals from five sites from our population genetics analyses due to their distant geographic locations from each other and from the remaining sites (>100 km) or small sample sizes at each location (*n* < 4). The final genotype data for population genetics analyses contained 336 *M. yumanensis*. These 336 samples were grouped into 10 geographic populations for the population genetics analyses, each with at least 20 individuals (Table 1). Seven geographic populations were located in southwest relatively coastal British Columbia: Alice Lake (AL), Colony Farm (CF), Deas Island (DI), Hayward Lake (HL), Stanley Park (SP), Stave Lake Lodge (SL), and Thompson Creek Farm (TC). Three were located in more interior British Columbia: Armstrong’s (AS), Condo (CD), and Lillooet (LO) (Figure 2). Details of the 10 geographic populations of *M. yumanensis* were presented in Table 1.

### 3.4. Allelic Diversities and Genotyping Error Estimates

Among the 11 microsatellite loci that we initially screened for *M. yumanensis*, two were excluded: Mluc21 had low amplification success, and Mluc5 was monomorphic in the 336 samples. This left nine polymorphic loci for downstream analyses. Allelic diversities and genotyping error estimates were then performed on the remaining nine microsatellite loci on 336 individuals (Mluc5 was included in the calculations of the allelic diversities, genotyping error estimates, tests of Hardy-Weinberg equilibrium and of linkage disequilibrium, but the results of Mluc5 was not shown since it was not monomorphic) (Table 2).

The nine microsatellite loci were designed based on the sequences of *M. lucifugus,* and all nine microsatellite loci were reported to have a motif size of 4 bp. However, motif size varied from those reported previously in the nine microsatellites when used to amplify loci in *M. yumanensis* (Table 2): alleles for six of the loci varied by 2 bp. Interestingly, three of these six loci (Mluc11, Mluc29, and Mluc34) showed motif size variations of 2 bp and 4 bp in *M. lucifugus* in the study by Burns et al. [24]. The number of unique alleles at each locus in both the total sample and in each geographic population was shown in Figure S1. The expected and observed heterozygosity at each locus in both the total sample and in each geographic population was shown in Figure S2. Together, Figures S1 and S2 demonstrated high microsatellite allelic and genetic diversity. Among the nine loci, Mluc30 had the highest number of unique alleles. Mluc30 and Mluc34 showed the highest observed heterozygosity, while Mluc29 showed the lowest observed and expected heterozygosity.

### 3.5. Hardy–Weinberg Equilibrium and Linkage Disequilibrium

Hardy–Weinberg equilibrium was first tested using all *M. yumanensis* from all geographic populations (*n* = 336). Complete results with all corrected *p*-values and fixation index (*F*_IS_) estimates were presented in Tables S1–S4. In the total population, the null hypothesis of Hardy–Weinberg equilibrium was rejected at two of the nine loci (Mluc8 and Mluc30). Further analyses revealed that the departure from Hardy–Weinberg equilibrium at Mluc30 was due to an overall heterozygote deficiency. Interestingly, though the overall genotype frequencies did not deviate significantly from Hardy–Weinberg equilibrium at locus Mluc11, there was a significant heterozygote excess for some of the allelic combinations. Because the Wahlund effect could cause deviation from the Hardy–Weinberg equilibrium in the total population even if each geographic population was in Hardy–Weinberg equilibrium, we also tested Hardy–Weinberg equilibrium within each of 10 geographic populations. The null hypothesis of Hardy–Weinberg equilibrium was rejected at Mluc4 in Lillooet (LO) (Table S2), where there was significant heterozygote deficiency (Table S3). Similarly, the null hypothesis of Hardy–Weinberg equilibrium was rejected at Mluc30 in Deas Island (DI) and Stave Lake Lodge (SL) sites (Table S2); significant heterozygote deficiency was found at this locus in the Armstrong’s (AS), Deas Island (DI), and Stave Lake Lodge (SL) populations (Table S3). In contrast, significant heterozygote excess was detected in the Lillooet (LO) population at Mluc8 (Table S4). No locus was consistently out of Hardy–Weinberg equilibrium in all geographic populations.

Linkage disequilibrium analyses between all 36 pairs of the 9 microsatellite loci for the total population of all *M. yumanensis* (*n* = 336) showed significant departure from linkage disequilibrium only between loci Mluc8 and Mluc30 (Figure S3a). Because admixture among geographic populations could induce linkage disequilibrium when genetically differentiated populations were treated as one population, we also tested linkage disequilibrium within each of the 10 geographic populations. Although linkage disequilibrium was detected between one pair of loci in the total population, within each of the 10 geographic populations, no pairs of loci showed significant departure from linkage disequilibrium (Figure S3b–k).

### 3.6. Population Structure in M. yumanensis

**Figure 3:**
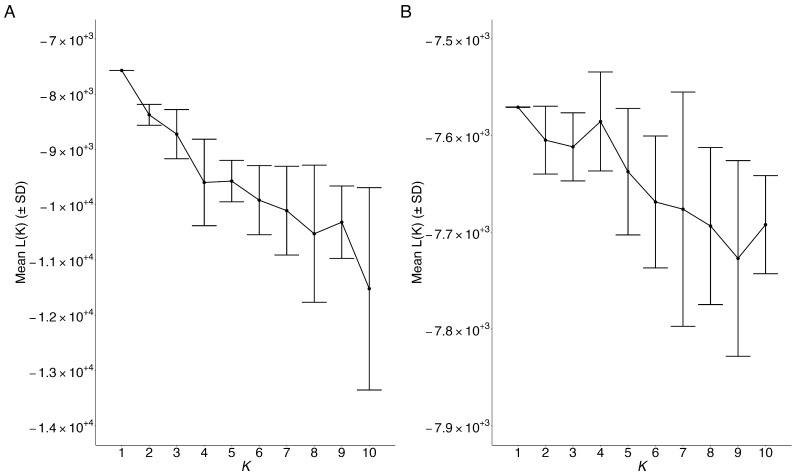
STRUCTURE results of 336 *M. yumanensis*. Mean log likelihood with standard deviation over 15 replicate runs of STRUCTURE at each user-defined number of genetic populations (*K*). (**A**) STRUCTURE model using only genetic information; (**B**) STRUCTURE model combined with LOCPRIOR model.

In the clustering results from STRUCTURE, the maximum mean log probability of the data were at K = 1 (Figure 3A), supporting the assumption that all 336 *M. yumanensis* belonged to the same metapopulation (species). Similarly, using geographic populations as prior information to help the clustering of individuals also only found one population in the 336 *M. yumanensis* (Figure 3B).

In AMOVA, 80 *M. yumanensis* were removed because of missing genotype data at one or more loci (individuals with greater than 5% missing data were removed by default). Although the among-population variance only represented about 2.8% of the total variance, the contribution was statistically significant (*p*-value = 0, Table 3).

**Figure 4:**
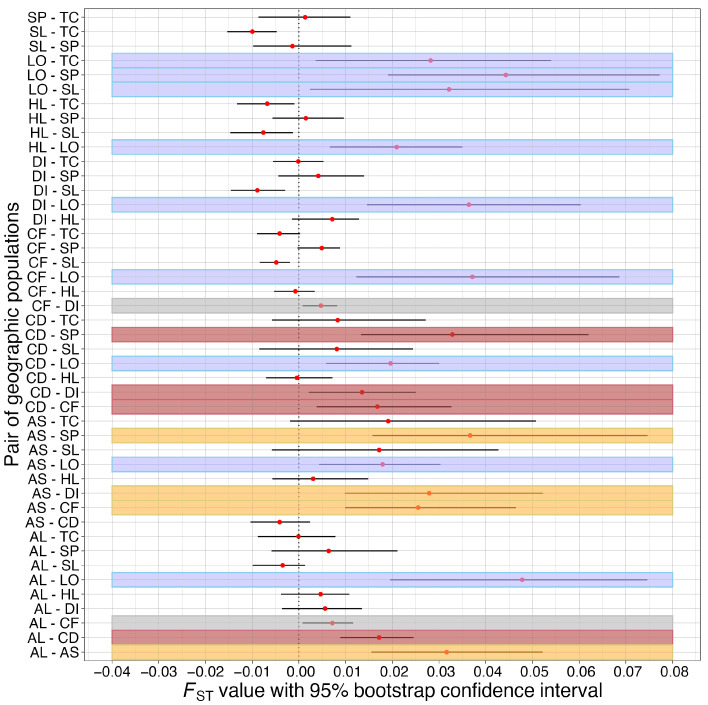
*F*_ST_ estimates between all pairs of geographic populations with 95% bootstrap confidence intervals. Larger *F*_ST_ values reflect greater genetic differentiation between the pair of geographic populations. AL = Alice Lake; AS = Armstrong’s; CD = Condo; CF = Colony Farm; DI = Deas Island; HL = Hayward Lake; LO = Lillooet; SL = Stave Lake Lodge; SP = Stanley Park; TC = Thompson Creek Farm. Population pairs with 95% confidence interval not overlapping with zero were labeled in different colors.

We next checked whether pairs of geographic populations were genetically differentiated from one another using pairwise *F*_ST_. All 45 pairs of geographic populations had *F*_ST_ estimates less than 0.05, with greater than half of them having a 95% confidence interval of their *F*_ST_ estimates overlapping with 0 (Figure 4). This result was consistent with the low overall differentiation detected by AMOVA. However, 19 of the 45 pairs of geographic populations (in different colors) had their 95% confidence intervals not overlapping with and greater than 0, suggesting that these 19 pairs of geographic populations were statistically differentiated. Among these 19 pairs of genetically differentiated geographic populations, 9 pairs involved the Lillooet (LO) population (blue color), 4 involved the Condo (CD) (red color, between the CD population and populations other than LO), 4 involved Armstrong’s (AS) (yellow color, between the AS and populations other than LO population), and 2 involved Colony Farm (CF) (grey color, between the CF and populations other than LO, CD, and AS populations). Overall, the Lillooet population had a higher genetic differentiation (*F*_ST_) from all other geographic populations than those among the remaining nine geographic populations.

### 3.7. Isolation-by-Distance and Isolation-by-Landscape Connectivity Test Results

**Figure A2:**
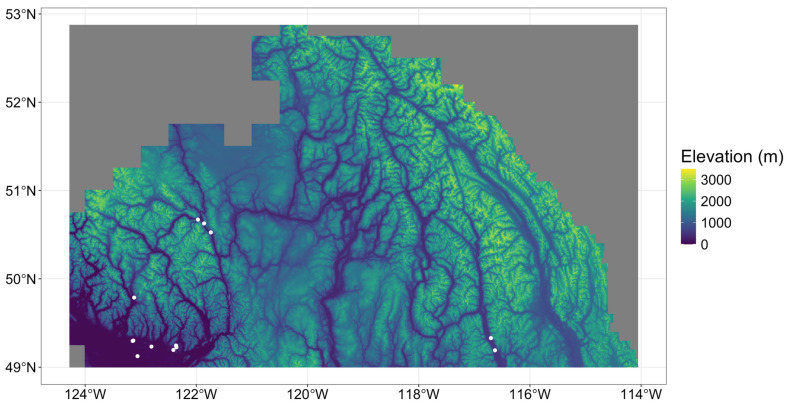
1:250,000 digital elevation model for British Columbia showing the elevation of areas of interest in southern British Columbia. White circles represented sampling sites. The digital elevation model was made using the cded_terra function in bcmaps R package (Version 2.2.0) [44]. The map was plotted using the tidyterra R package (Version 0.7.0) [66].

The isolation-by-distance model was significant (Mantel statistic r = 0.7046, *p*-value = 0.003), meaning that neighboring geographic populations were overall more genetically similar to each other than distant geographic populations. Although the isolation-by-landscape connectivity model that included elevation information (Figure A2) was also significant (Mantel statistic r = 0.5688, *p*-value = 0.018), there was a weaker correlation.

### 3.8. Detection of P. destructans

We did not detect the fungal pathogen *P. destructans* in any of the 336 wing swabs sampled from across all 10 geographic areas in this study.

## 4. Discussion

This study investigated the patterns of genetic variation in populations of *M. yumanensis* from Western Canada. Our analyses identified that nine microsatellite markers originally developed for *M. lucifugus* could help distinguish between *M. lucifugus* and *M. yumanensis*. In addition, these genetic markers revealed high allelic and genotypic diversities within most geographic populations of *M. yumanensis*. Our population genetics analyses revealed that *M. yumanensis* in Western Canada belonged to one largely randomly mating meta-population, with limited genetic differentiation among coastal populations and low but statistically significant genetic differentiation among some inland populations and between a few inland and coastal populations. Both geographic distance and landscape features showed significant influences on genetic relationships among populations of *M. yumanensis.* Interestingly, despite its presence in the neighboring Washington State in the United States of America since 2016, WNS fungal pathogen *P. destructans* was not detected in the wing swabs of any of our sampled individuals. However, despite its absence in our bat wing swabs, the fungal pathogen was reported in bat guano in Grand Forks, southern British Columbia, in 2023. Given the mass decline in North American bat populations caused by WNS and the susceptibility of *M. yumanensis* to WNS, knowledge about their populations will help future conservation of this species from this disease.

Overall, our investigation of population structure using 9 nuclear microsatellite loci on 10 geographic populations of *M. yumanensis* in British Columbia, Western Canada, detected weak but statistically significant population structure, rejecting the null hypothesis of no genetic differentiation among populations. Although our STRUCTURE analysis detected only one genetic cluster in our *M. yumanensis* samples (Figure 3), AMOVA and pairwise *F*_ST_ showed small but significant genetic differentiation among several geographic populations (Table 3, Figure 4). Specifically, several pairs of geographic populations had significant *F*_ST_ values with their 95% bootstrap confidence intervals not overlapping with 0 (Figure 4). Significant *F*_ST_ values were mostly found in pairs of populations that included Lillooet (LO), Armstrong’s (AS), and Condo (CD), the three populations most distant from the other geographic populations, supporting our finding of genetic isolation-by-geographic distance. Two factors might have contributed to these observations. In the first, there was a wide range of geographic distances between sites, ranging from about 2 km to 470 km (Table S5), and dispersal over long distances might be rare. Second, most sampled sites were maternity colonies of adult females that roost together each summer with high rates of fidelity to their natal roost [53]. Interestingly, including elevation in the Mantel test did not improve the prediction of how geographic factors influenced the genetic pattern, suggesting that this species may fly to high elevations when migrating/breeding. This is consistent with the findings by one of us (C.L.L.) that this species is acoustically detected at high elevations and has been radio-tracked to a roost at 1800 m above sea level [21].

One interesting finding from the genetic data is that the Lillooet (LO) population was most differentiated from other populations despite Armstrong’s (AS) and Condo’s (CD) being more distantly located from the other seven coastal populations. The results suggest that there might be other factors contributing to the differentiation of the Lillooet (LO) population in addition to geographic distance, such as localized adaptation and geographically restricted interbreeding [54,55]. Although we tend to think of rivers as likely flight corridors for bat movement for breeding/hibernation/migration, this is not the case for all species [56]. The Fraser River seems like a likely conduit of gene flow between Lillooet and Vancouver region populations, but our results reject that hypothesis. *M. yumanensis* is confined to areas of relatively mild winter climates and has been documented overwintering in buildings used as maternity roosts in some areas of southern British Columbia [21]. Ranging as far north as southeastern Alaska along the coast [10], this species is not typically found in the northern half of British Columbia [21] or on the eastern side of the Rocky Mountains, where winters are more extreme [57]. Lillooet is the least snowy of our sampling sites and the hottest. In fact, Lillooet-Lytton is one of the hottest places in Canada in the summer; in July 2023, Lillooet had the highest average daytime temperature in Canada, around 34.0 °C [58], and in June 2021, during a heat dome in British Columbia, the daytime highest temperature of this Lytton-Lillooet area was about 50 °C, setting Canadian records [59]. It is possible that certain adaptations could mold gene flow and contribute to ecological isolation among some bat populations.

The weak population structure found by the microsatellite genotype data among the populations across southern British Columbia could be due to two non-mutually exclusive explanations: (1) there is extensive gene flow among those populations; (2) there is a lack of power of these microsatellite markers to distinguish populations, causing underestimation of genetic differentiation. The first explanation was supported by acoustic records, which have shown patterns indicative of some degree of seasonal migration of *M. yumanensis* among regions in Southwestern British Columbia [21]. Although the high number of alleles and high allelic diversity at most loci may suggest that the second explanation is unlikely a contributor to the observed low genetic differentiation, limitations of microsatellite loci may still underestimate the real genetic differentiation. For example, homoplasy would cause microsatellite alleles with different DNA sequences but the same length to be treated as the same allele, thus underestimating the variation between alleles [60]. Confirmation of whether homoplasy underestimates the allelic diversity requires sequencing the putatively homoplasic alleles or using denaturing gel electrophoresis, such as single-strand conformation polymorphisms, to separate such alleles [60]. Another limitation is that the upper bound of *F*_ST_ increases when the frequency of the most frequent allele approaches 0.5. Microsatellite loci tend to show lower *F*_ST_ than biallelic single nucleotide polymorphisms since microsatellite loci tend to have many alleles at each locus, causing lower frequency for each allele (less than 0.5) in general and thus a lower upper bound of *F*_ST_ [61]. In our study, allele frequencies of microsatellite loci showed only a few loci with the dominant allele close to 0.5 in some geographic populations, which might contribute to the lower maximum value of *F*_ST_ and thus lower *F*_ST_ estimates (Figures S4–S14). Together, such limitations of microsatellite markers might cause lower than actual genetic differentiation (low *F*_ST_) of *M. yumanensis* populations and uncertain genetic clustering of some individuals. For example, STRUCTURE results found one potential admixed individual assigned to neither *M. yumanensis* nor *M. lucifugus* and some inconsistent clustering between field identification and STRUCTURE clustering (Figure 1 and Figure A1). Targeted multilocus DNA sequencing or genome-wide single nucleotide polymorphisms based on whole-genome sequencing will help resolve the issues about levels of population genetic differentiation and clarify the genetic identity of the uncertain individuals. The recently published whole-genome sequence of *M. yumanensis* [18], along with that published earlier for *M. lucifugus* [62], could serve as excellent reference sequences for such comparisons. Similarly, the whole-genome sequence of *M. yumanensis* [18] could serve as a reference sequence for population genomic analyses of *M. yumanensis*, including critically evaluating the relationships among the six subspecies of *M. yumanensis*: *M. y. lambi* Benson, 1947*; M. y. lutosus* Miller and G. M. Allen, 1928*; M. y. oxalis* Dalquest, 1947*; M. y. saturatus* Miller, 1897*; M. y. sociabilis* H. W. Grinnell, 1914*;* and *M. y. yumanensis* H. Allen, 1864 [11].

Similar studies have been performed for various populations of *M. lucifugus*. For example, range-wide population genetics analysis of *M. lucifugus* across North America by Vonhof et al. showed evidence for genetic isolation by geographic distance based on microsatellite loci across both the whole range as well as across Western North America (including British Columbia). Interestingly, the *F*_ST_ values among Western North American *M. lucifugus* populations ranged between 0.002 and 0.018, and those among eastern North American samples ranged between −0.005 and 0.013 [3], both of which were smaller than those between many geographic populations of *M. yumanensis* in southern British Columbia found in this study. In another range-wide study on the northern range of *M. lucifugus* by Davy et al., three major genetic populations were identified, with admixture individuals between contact zones: an eastern population ranging from Prince Edward Island to Manitoba, a Montane Cordillera population that included those from southern British Columbia, and a Haida Gwaii population. Their analyses revealed that neither the Rocky Mountains nor the Coastal Mountains acted as a barrier to gene flow within *M. lucifugus* [9]. These results on *M. lucifugus* were similar to ours on *M. yumanensis* from Western Canada, where STRUCTURE analyses revealed that all samples from southern British Columbia belonged to one large meta-population, despite the presence of several mountains within our sampled region (Figure 3). However, *M. yumanensis* has only been reported on the western side of the Continental Divide, suggesting that the Rocky Mountains is likely a significant barrier for the dispersal of *M. yumanensis*, different from those observed for *M. lucifugus* [3,9].

We did not detect the fungal pathogen *P. destructans* in any of the analyzed *M. yumanensis* bat wing swabs, rejecting our hypothesis of the presence of *P. destructans* in our samples. However, this lack of detection does not mean that the fungal pathogen is not in British Columbia or that these bats in British Columbia will not be affected or killed by *P. destructans* in the future. Indeed, the fungal pathogen has been reported from bat guano samples in Grand Forks, indicating its presence in British Columbia. In addition, in neighboring Washington State, to the south of British Columbia, *P. destructans* infection and WNS have been reported for *M. yumanensis* bats [14]. It’s highly likely that the fungus will be found on bats in British Columbia very soon.

For the Little Brown Bat (*M. lucifugus*), a comparison between populations captured before and after the arrival of WNS showed either no difference [63] or evidence for a difference due to adaptation to WNS and/or genetic drift between those populations [9,64]. For example, notable allele frequency differences were observed between the pre-WNS and post-WNS populations in genes associated with regulating arousal from hibernation (GABARB1), breakdown of fats (cGMP-PK1), and vocalizations (FOXP2) [64]. In contrast, there was a limited difference between the populations at the DRB1-like exon 2 of the major histocompatibility complex in *M. lucifugus.* However, interpretation of the DRB1-like exon 2 data was confounded by the duplication of the DRB1 locus [9]. Correctly assigning alleles to the corresponding duplicated loci is required for further testing the immunogenetic selection driven by the WNS [9]. These findings suggested a potential relationship between the WNS and the genetic characteristics of the bat populations. At present, almost nothing is known about the potential genetic relationships between *M. yumanensis* and their susceptibility to the WNS fungal pathogen *P. destructans*. Continuing monitoring of the population trend and infection status of the *M. yumanensis* is required, not only to protect the *M. yumanensis* colonies but also to protect the co-roosting *M. lucifugus*.

The decline of bat populations has shown to have profound consequences for agricultural pest management. In addition, while the fungal pathogen cannot directly infect and cause diseases in humans, the reduced bat populations have led to significantly increased insecticide usage in the eastern United States of America, which was correlated with higher human infant mortality [1]. Together, such results emphasized the importance of population studies of both the hosts and the pathogens in order to maintain and protect a healthy ecosystem for human well-being [65].

## 5. Conclusions and Perspectives

In conclusion, microsatellite genotypes from 336 *M. yumanensis* in British Columbia, Western Canada, showed an overall low but statistically significant population structure with more genetic differentiation found between populations in interior British Columbia and the coastal populations than among coastal populations. Interestingly, the observed genetic differentiations among geographic populations of *M. yumanensis* in southern British Columbia were overall higher than those of *M. lucifugus* populations from more distant geographic regions across most of North America. Such a result suggests that gene flow is more prevalent among geographic populations of *M. lucifugus* than those of *M. yumanensis.* Because these two species often roost together in British Columbia and both are susceptible to WNS, we hypothesize that between the two bat species, *M. lucifugus* is more likely to bring in and spread the fungal pathogen than *M. yumanensis.* Though no evidence of *P. destructans* was found on the wings of the sampled *M. yumanensis* in 2022 and 2023 in southern British Columbia, continued surveillance of the fungal pathogen in both *M. lucifugus* and *M. yumanensis* is needed to test this hypothesis and to closely monitor the emergence and potential spread of the disease in this region.

## Figures and Tables

**Table 1:** Names and sample sizes of the 10 geographic populations and corresponding sampling sites of *M. yumanensis* analyzed in this study. For the geographic populations with multiple sampling sites, such as Lillooet and Stanley Park, the mean latitude and longitude were used as the geographic coordinates for that geographic population.

Geographic Population ^1^	Sample Size	Sampling Site ^2^	Type of Sampling Site
Alice Lake (AL)	56	Alice Lake	Maternity roost
Armstrong’s (AS)	30	Armstrong’s	Maternity roost
Condo (CD)	23	Condo	Maternity roost
Colony Farm (CF)	74	Colony Farm	Maternity roost
Deas Island (DI)	35	Deas Island	Maternity roost
Hayward Lake (HL)	24	Hayward Lake	Maternity roost
Lillooet (LO)	26	South Lillooet	Foraging location
		Central Lillooet	Foraging location
		West Lillooet	Foraging location
Stave Lake Lodge (SL)	21	Stave Lake Lodge	Maternity roost
Stanley Park (SP)	24	Stanley Park 1	Foraging location
		Stanley Park 2	Foraging location
Thompson Creek Farm (TC)	23	Thompson Creek Farm	Foraging location
Total number of samples	336		

^1^ Geographic population: manually defined based on consideration of geographic distances, type of sampling sites, and sample sizes for population genetics analysis. Hayward Lake and Stave Lake Lodge were treated as separate populations because they were different maternity roosts, and we would like to check whether there was genetic differentiation between the two sampling sites. The 3 sampling sites in Lillooet were treated as one population because Central Lillooet (*n* = 1) and South Lillooet (*n* = 3) contained too few individuals. ^2^ Sampling site: Geographic location where the bats were captured.

**Table 2:** Expected and observed motif size in base pairs (bp), number of unique alleles, observed and expected heterozygosity, polymorphism information content, mean error rate per allele, and mean error rate per locus at the nine polymorphic nuclear microsatellite loci of 336 *M. yumanensis* in British Columbia, Western Canada.

Locus	Expected/Observed Motif Size (bp) ^1^	Number of Unique Alleles	Heterozygosity (Observed/Expected) ^2^	PIC ^3^	Mean Error Rate (Per Allele/Per Locus) ^4^
Mluc4	4/4	3	0.34/0.39	0.31	0.0315/0.0631
Mluc25	4/2	11	0.69/0.73	0.69	0.0096/0.0192
Mluc34	4/2	11	0.84/0.83	0.81	0.0000/0.0000
Mluc8	4/4	17	0.83/0.79	0.77	0.0860/0.1627
Mluc11	4/2	4	0.4/0.36	0.32	0.0050/0.0100
Mluc29	4/2	4	0.03/0.03	0.03	0.0076/0.0152
Mluc1	4/4	4	0.37/0.36	0.32	0.0000/0.0000
Mluc7	4/2	10	0.81/0.81	0.79	0.0000/0.0000
Mluc30	4/2	28	0.84/0.94	0.94	0.1111/0.1111

^1^ Expected motif size was the size of the repeat motif of all nine microsatellite loci as reported in Burns et al. [24]; the observed motif size was the smallest difference between allele sizes after allele binning in our study. ^2^ Observed heterozygosity was the proportion of individuals that are heterozygous; expected heterozygosity was the expected proportion of heterozygous individuals based on Hardy–Weinberg expectation. ^3^ Polymorphism information content (PIC) was a measure of polymorphism of molecular markers based on Botstein et al.; Loci with the PIC value greater than 0.5 were considered highly polymorphic [52]. ^4^ Mean error rate per allele and mean error rate per locus calculated based on Pompanon et al. [37].

**Table 3:** Analysis of molecular variance (AMOVA) using the poppr.amova function in poppr R package.

Source of Variation (df *)	Sums of Square Deviations	Mean Square Deviations	Variance Components (Percent Relative to Total)	*p*-Value
Population (df = 9)	40.38601	4.487335	0.07570628 (2.81898%)	0
Error (df = 245)	639.42183	2.609885	2.60988502 (97.18102%)	
Total (df = 254)	679.80784	2.676409		

* Degrees of freedom.

## Data Availability

All genotype data of *M. yumanensis* generated and reported for this study are presented in the manuscript.

## Supplementary Materials

The following supporting information can be downloaded at: https://www.mdpi.com/article/10.3390/ani15040578/s1. File S1: 336_microsatellite_genotypes.csv: Microsatellite genotypes of the 336 *M. yumanensis*; File S2: clean_electropherogram_files.zip: electropherogram files of the microsatellite genotypes after removing output files with no results, potential contamination, and so on; File S3: genepop_hardy_weinberg_results.zip: raw data from Genepop analyses of Hardy-Weinberg equilibrium; File S4: genepop_linkage_disequilibrium_results.zip: raw data from Genepop analyses of linkage disequilibrium; File S5: MYYU_Population_Structure.Rmd: R codes for analyses and generating figures; File S6: replicate_genotypes.csv: replicated samples for estimating genotyping errors; File S7: structure_results.zip: raw data from STRUCTURE analyses on the 336 *M. yumanensis*; File S8: supplementary_data.docx: supplementary figures (Figures S1–S14) and tables (Tables S1–S5).

## Appendix A

**Table A1:** Reagents and their volumes for preparing the three multiplex PCRs for amplifying the 11 nuclear microsatellite markers.

Reagents *	Volume Per Reaction	Final Concentration
Multiplex 1		
Double-Distilled Water	4.8 μL	-
5 X Colorless GoTaq Flexi Buffer	2 μL	1 X
Magnesium Chloride (25 mM)	0.6 μL	1.5 mM
Deoxynucleotide (dNTP) Solution Mix (10 mM each dNTP)	0.2 μL	0.2 mM
Mluc4 Forward Primer (10 μM)	0.3 μL	0.3 μM
Mluc4 Reverse Primer (10 μM)	0.3 μL	0.3 μM
Mluc25 Forward Primer (10 μM)	0.2 μL	0.2 μM
Mluc25 Reverse Primer (10 μM)	0.2 μL	0.2 μM
Mluc34 Forward Primer (10 μM)	0.15 μL	0.15 μM
Mluc34 Reverse Primer (10 μM)	0.15 μL	0.15 μM
GoTaq G2 Hot Start Taq Polymerase (5 u/μL)	0.1 μL	0.05 u/μL
DNA	1 μL	-
Total	10 μL	-
Multiplex 2		
Double-Distilled Water	4 μL	-
5 X Colorless GoTaq Flexi Buffer	2 μL	1 X
Magnesium Chloride (25 mM)	0.6 μL	1.5 mM
Deoxynucleotide (dNTP) Solution Mix (10 mM each dNTP)	0.2 μL	0.2 mM
Mluc5 Forward Primer (10 μM)	0.25 μL	0.25 μM
Mluc5 Reverse Primer (10 μM)	0.25 μL	0.25 μM
Mluc8 Forward Primer (10 μM)	0.15 μL	0.15 μM
Mluc8 Reverse Primer (10 μM)	0.15 μL	0.15 μM
Mluc11 Forward Primer (10 μM)	0.35 μL	0.35 μM
Mluc11 Reverse Primer (10 μM)	0.35 μL	0.35 μM
Mluc29 Forward Primer (10 μM)	0.3 μL	0.3 μM
Mluc29 Reverse Primer (10 μM)	0.3 μL	0.3 μM
GoTaq G2 Hot Start Taq Polymerase (5 u/μL)	0.1 μL	0.05 u/μL
DNA	1 μL	-
Total	10 μL	-
Multiplex 3		
Double-Distilled Water	3.5 μL	-
5 X Colorless GoTaq Flexi Buffer	2 μL	1 X
Magnesium Chloride (25 mM)	0.6 μL	1.5 mM
Deoxynucleotide (dNTP) Solution Mix (10 mM each dNTP)	0.2 μL	0.2 mM
Mluc1 Forward Primer (10 μM)	0.3 μL	0.3 μM
Mluc1 Reverse Primer (10 μM)	0.3 μL	0.3 μM
Mluc7 Forward Primer (10 μM)	0.25 μL	0.25 μM
Mluc7 Reverse Primer (10 μM)	0.25 μL	0.25 μM
Mluc21 Forward Primer (10 μM)	0.45 μL	0.45 μM
Mluc21 Reverse Primer (10 μM)	0.45 μL	0.45 μM
Mluc30 Forward Primer (10 μM)	0.3 μL	0.45 μM
Mluc30 Reverse Primer (10 μM)	0.3 μL	0.3 μM
GoTaq G2 Hot Start Taq Polymerase (5 u/μL)	0.1 μL	0.05 u/μL
DNA	1 μL	-
Total	10 μL	-

* Company names of the reagents: Deoxynucleotide (dNTP) Solution Mix (Catalog # N0447L, New England Biolabs , Ipswich, MA, USA), Primers (Integrated DNA Technologies, Coralville, IA, USA), 5 X Colorless GoTaq Flexi Buffer, Magnesium Chloride, and GoTaq G2 Hot Start Taq Polymerase (Catalog number: M7405) (Promega, Madison, WI, USA).

**Table A2:** Primers and fluorescent dyes and PCR conditions for amplifying the 11 nuclear microsatellite loci.

Locus	Primer Sequence	Reference	PCR Condition
Multiplex 1			Initial denaturation at 95 °C for 5 min 35 cycles of denaturation at 95 °C for 30 s, annealing at 56 °C for 1 min, extension at 72 °C for 1 min Final extension at 64 °C for 45 min Held at 4 °C after completion
Mluc4	F: 5′-/5HEX/AACTAACCGAGCTACCCAATCA-3	Burns et al. [24]	
	R: 5′-CTTTCCTTTCTCCCTTCCACTC-3′		
Mluc25	F: 5′-/56-FAM/TACACCCTCTCCAGTTCATGTG-3′	Burns et al. [24]	
	R: 5′-GAGATTACCATAGGCTCACCAAA-3′		
Mluc34	F: 5′-/5ATTO550N/ACAAAACACATAGATCCACCCC-3′	Burns et al. [24]	
	R: 5′-GCCAACTTCAAAGAGAAAGGAA-3′		
Multiplex 2			
Mluc5	F: 5′-/5HEX/CTAAGAAAGGGTTGCACTCTGG-3′	Burns et al. [24]	
	R: 5′-TTGTTTACATCAGGCTTTGTGC-3′		
Mluc8	F: 5′-/5ATTO550N/CCACTCAAGCACCAGATGAATA-3′	Burns et al. [24]	
	R: 5′-AGGAATGAGGGAAGAAAGGAAG-3′		
Mluc11	F: 5′-/5ATTO565N/CATAAGCTGAATGAGAGGAGGG-3′	Burns et al. [24]	
	R: 5′-TCGAATAAATACCTGGGAATGG-3′		
Mluc29	F: 5′-/56-FAM/GGAGGTGGAGAGATTGAGAAAA-3′	Burns et al. [24]	
	R: 5′-GACACAATGAAGTCCCAAACAA-3′		
Multiplex 3			
Mluc1	F: 5′-/5HEX/ATCATAGCGAGCGATCAAACTC-3′	Burns et al. [24]	
	R: 5′-GCTCTTCTTTTGGTACAGGTGG-3		
Mluc7	F: 5′-/5ATTO550N/AATACCCTTGCCTTTCTTCCTC-3′	Burns et al. [24]	
	R: 5′-ATGTTTTCCTCAAAGTCCCTCA-3′		
Mluc21	F: 5′-/56FAM/CACTGGTATAGTTCTTTGTAGGTCTG-3′	Burns et al. [24]	
	R: 5′-AATTTGAATGCTATGGCGAC-3′		
Mluc30	F: 5′-/5ATTO565N/CACACACACAACAGAGAGAGAGAG-3′	Burns et al. [24]	
	R: 5′-AAAAGCTGGAAAGAAACACTGC-3′		
