# Supplementary material for: High Diversity and Low Genetic Differentiation Among Geographic Populations of Myotis yumanensis in Western Canada
Source: Animals (Basel). 2025 Feb 18;15(4):578. doi: 10.3390/ani15040578 (PMC11851726; doi:10.3390/ani15040578)
Supplement: Supplementary file 1 [file animals-15-00578-s001.zip › supplementary_file.pdf]

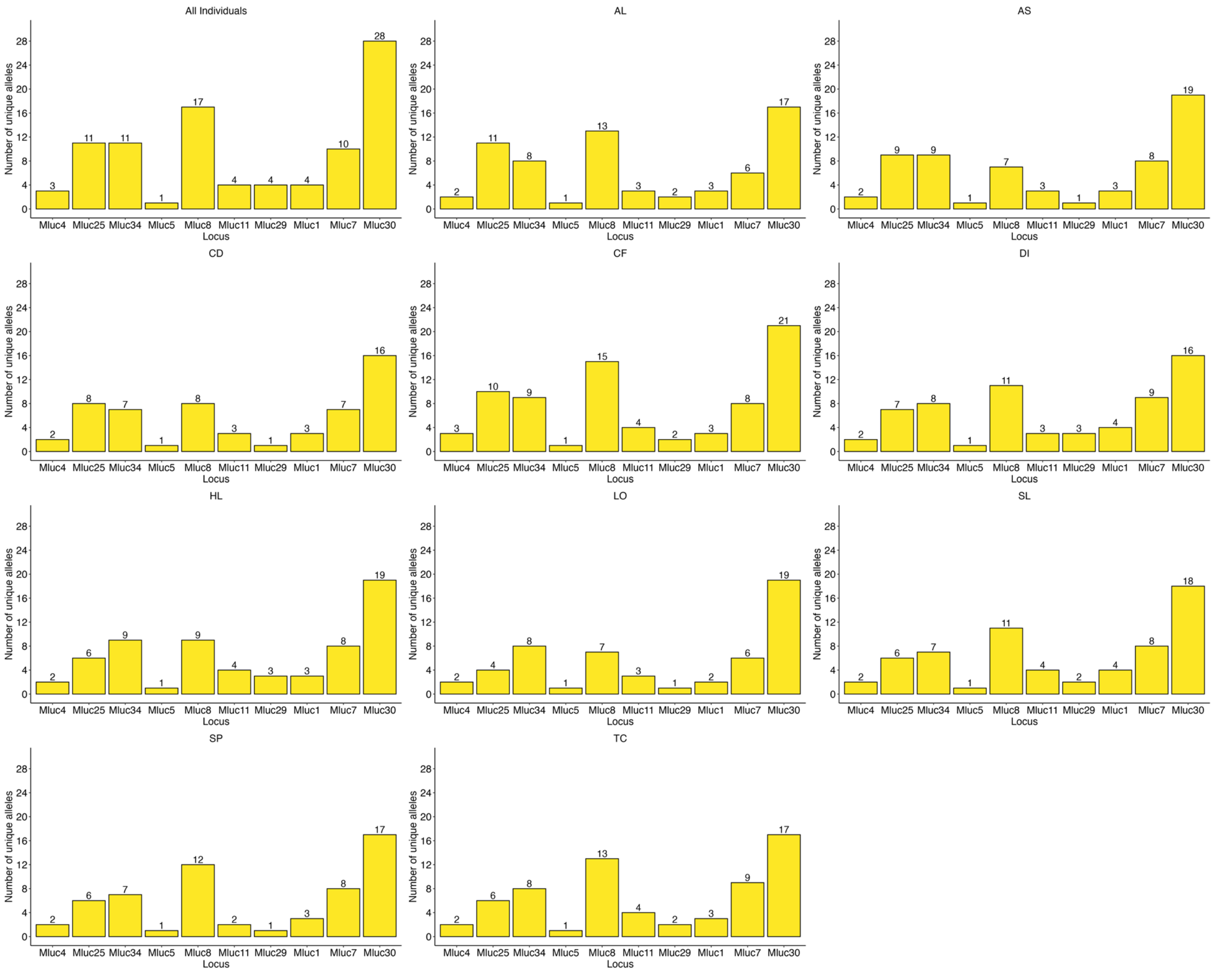

Figure S1. Number of unique alleles in all individuals and in each geographic population. AL = Alice Lake; AS = Armstrong's; CD = Condo; CF = Colony Farm; DI = Deas Island; HL = Hayward Lake; LO = Lillooet; SL = Stave Lake Lodge; SP = Stanley Park; TC = Thompson Creek Farm. *Mluc*5 was monomorphic and was removed from downstream analyses.

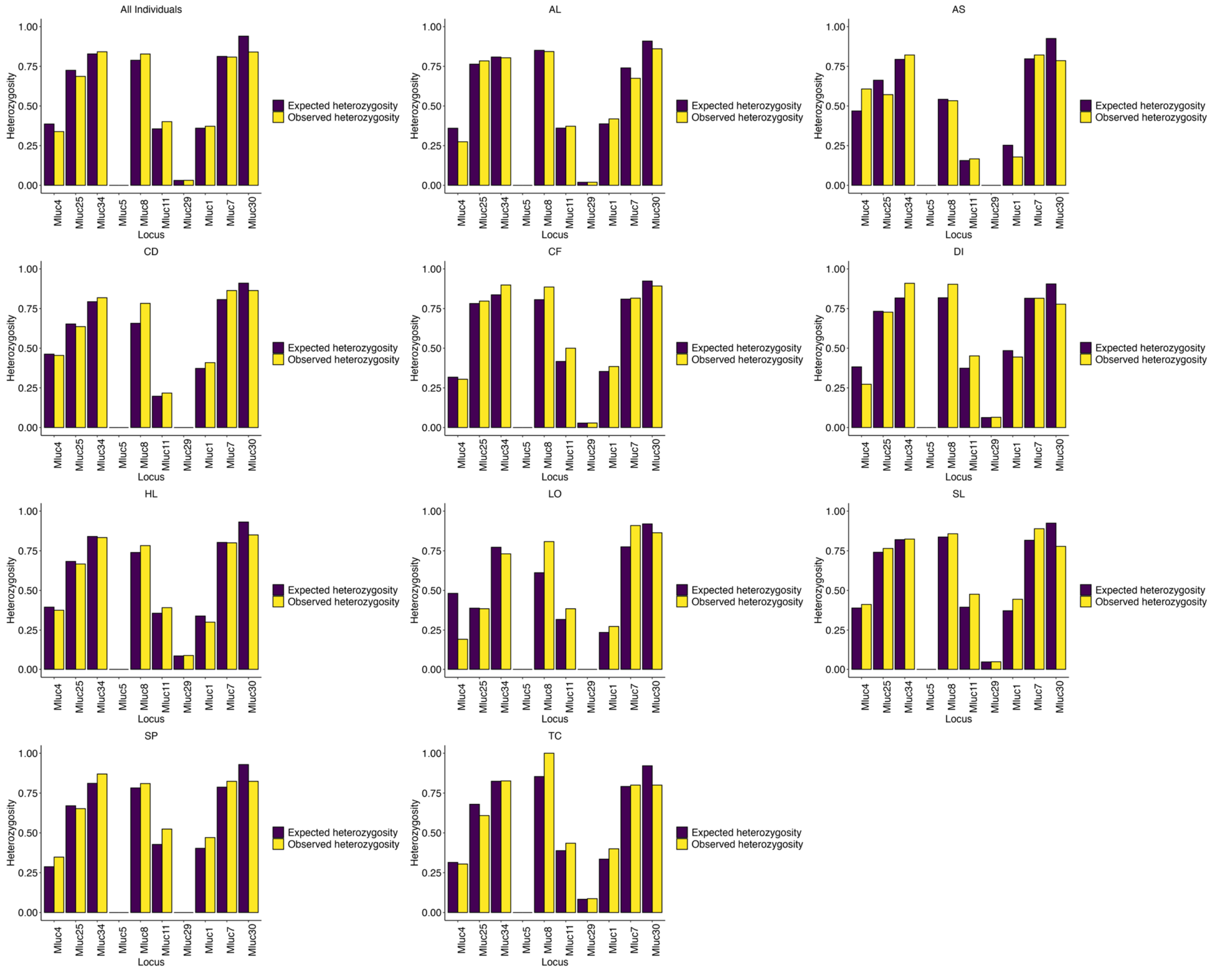

Figure S2. Expected and observed heterozygosity in all individuals and in each geographic population. AL = Alice Lake; AS = Armstrong's; CD = Condo; CF = Colony Farm; DI = Deas Island; HL = Hayward Lake; LO = Lillooet; SL = Stave Lake Lodge; SP = Stanley Park; TC = Thompson Creek Farm. *Mluc*5 was monomorphic and was removed from downstream analyses.

Table S1. Tests of Hardy-Weinberg equilibrium, heterozygote deficiency, and heterozygote excess for the 9 microsatellite loci in all individuals using Genepop program (Version 4.8.3). P-values were corrected for multiple comparisons within each test using the `p.adjust` function with Benjamini and Hochberg method in `stats` R package. WC was the Weir and Cockerham's estimator of F_{IS}. RH was the Robertson and Hill's estimator of F_{IS}. Results for *Mluc*5 was not included because it was monomorphic and not informative.

	**Hardy-Weinberg Equilibrium**	**Heterozygote Deficiency**	**Heterozygote Excess**	F_{IS}	
**Locus**	**Corrected *P***	**Corrected *P***	**Corrected *P***	**WC**	**RH**
*Mluc*4	0.1626	0.0990	1.0000	0.1258	0.0640
*Mluc*25	0.2894	0.3116	1.0000	0.0546	0.0167
*Mluc*34	0.6640	0.9890	1.0000	-0.0149	-0.0105
*Mluc*8	**0.0000**	0.3116	1.0000	-0.0479	0.0153
*Mluc*11	0.2754	1.0000	**0.0108**	-0.1255	-0.0630
*Mluc*29	1.0000	1.0000	1.0000	-0.0116	-0.0038
*Mluc*1	0.2894	0.5314	1.0000	-0.0317	0.0120
*Mluc*7	0.2894	0.7194	1.0000	0.0069	-0.0013
*Mluc*30	**0.0000**	**0.0000**	1.0000	0.1079	0.0626

Table S2. Tests of Hardy-Weinberg equilibrium in each geographic population using Genepop program (Version 4.8.3). P-values were corrected for multiple comparisons within each geographic population using the `p.adjust` function with Benjamini and Hochberg method in `stats` R package. WC was the Weir and Cockerham's estimator of F_{IS}. RH was the Robertson and Hill's estimator of F_{IS}. Results for *Mluc*5 was not included because it was monomorphic and not informative. AL = Alice Lake; AS = Armstrong's; CD = Condo; CF = Colony Farm; DI = Deas Island; HL = Hayward Lake; LO = Lillooet; SL = Stave Lake Lodge; SP = Stanley Park; TC = Thompson Creek Farm.

Locus	AL			AS			CD		
	**Corrected**	F_{IS}		**Corrected**	F_{IS}		**Corrected**	F_{IS}	
	P	**WC**	**RH**	***P***	**WC**	**RH**	***P***	**WC**	**RH**
*Mluc*4	0.3194	0.2465	0.2496	0.3622	-0.2786	-0.2822	1.0000	0.0411	0.0421
*Mluc*25	0.9735	-0.0170	-0.0179	0.3622	0.1554	0.0582	1.0000	0.0485	-0.0249
*Mluc*34	0.9735	0.0161	0.0057	0.3622	-0.0164	0.0226	1.0000	-0.0080	0.0116
*Mluc*8	0.3194	0.0192	0.0455	0.9495	0.0333	-0.0016	1.0000	-0.1699	-0.0625
*Mluc*11	0.3194	-0.0226	-0.0482	1.0000	-0.0507	-0.0290	1.0000	-0.0784	-0.0396
*Mluc*29	-	-	-	-	-	-	-	-	-
*Mluc*1	1.0000	-0.0686	-0.0609	0.3622	0.3095	0.1458	1.0000	-0.0739	-0.0966
*Mluc*7	0.6558	0.1004	0.0726	0.4333	-0.0122	-0.0100	1.0000	-0.0472	-0.0360
*Mluc*30	0.2376	0.0653	0.047	0.3622	0.1686	0.1177	1.0000	0.0742	0.0319

Locus	CF			DI			HL		
	**Corrected**	F_{IS}		**Corrected**	F_{IS}		**Corrected**	F_{IS}	
	P	**WC**	**RH**	***P***	**WC**	**RH**	***P***	**WC**	**RH**
*Mluc*4	0.9907	0.0486	0.0283	0.3118	0.3010	0.3070	1.0000	0.0717	0.0734
*Mluc*25	0.4736	-0.0123	-0.0219	0.8594	0.0229	0.0033	0.7664	0.0454	0.0626
*Mluc*34	0.9907	-0.0667	-0.0509	0.1499	-0.0978	-0.0303	1.0000	0.0295	0.0199
*Mluc*8	0.2379	-0.0916	-0.0376	0.8594	-0.0881	-0.0250	0.7596	-0.0366	-0.0138
*Mluc*11	0.7193	-0.1932	-0.0829	0.2574	-0.1915	-0.1239	0.7664	-0.0761	-0.0530
*Mluc*29	1.0000	-0.0073	-0.0074	1.0000	-0.0084	-0.0003	1.0000	-0.0115	-0.0005
*Mluc*1	0.9907	-0.0800	-0.0649	0.3118	0.1022	0.2295	0.5620	0.1396	0.2722
*Mluc*7	0.2379	0.0007	-0.0069	0.8594	0.0189	-0.0010	1.0000	0.0288	-0.0019
*Mluc*30	0.0720	0.0417	0.0177	**0.0153**	0.1594	0.1310	0.1188	0.1126	0.1023

Locus	LO			SL			SP		
	**Corrected**	F_{IS}		**Corrected**	F_{IS}		**Corrected**	F_{IS}	
	P	**WC**	**RH**	***P***	**WC**	**RH**	***P***	**WC**	**RH**
*Mluc*4	**0.0248**	0.6130	0.6326	1.0000	-0.0275	-0.0283	1.0000	-0.1892	-0.1926
*Mluc*25	0.5572	0.0291	-0.0480	1.0000	-0.0024	-0.0260	0.7888	0.0490	0.0023
*Mluc*34	0.5572	0.0732	0.0246	1.0000	0.0261	-0.0031	1.0000	-0.0501	-0.0625
*Mluc*8	0.5572	-0.3027	-0.0933	0.6509	0.0000	0.0396	0.8143	-0.0104	-0.0268
*Mluc*11	0.7257	-0.1933	-0.1014	1.0000	-0.1834	-0.0818	0.8143	-0.2022	-0.2061
*Mluc*29	-	-	-	-	-	-	-	-	-
*Mluc*1	1.0000	-0.1351	-0.1378	1.0000	-0.1674	-0.0686	0.5792	-0.1378	-0.1131
*Mluc*7	0.5572	-0.1507	-0.1127	0.6509	-0.0604	-0.0642	0.6029	-0.0159	-0.0301
*Mluc*30	0.2448	0.0838	0.0522	**0.0000**	0.1863	0.1505	0.2080	0.1434	0.1172

Locus	TC		
	**Corrected**	F_{IS}	
	P	**WC**	**RH**
*Mluc*4	1.0000	0.0552	0.0565
*Mluc*25	0.7320	0.1262	0.0783
*Mluc*34	0.7320	0.0199	-0.0114

*Mluc*8	0.8475	-0.1500	-0.0625
*Mluc*11	0.8475	-0.0973	-0.0649
*Mluc*29	1.0000	-0.0233	-0.0238
*Mluc*1	1.0000	-0.1692	-0.1020
*Mluc*7	0.8475	0.0146	0.0214
*Mluc*30	0.3510	0.1567	0.1268

Table S3. Tests of heterozygote deficiency in each geographic population using Genepop program (Version 4.8.3). P-values were corrected for multiple comparisons within each locus using the `p.adjust` function with Benjamini and Hochberg method in `stats` R package. WC was the Weir and Cockerham's estimator of F_{IS}. RH was the Robertson and Hill's estimator of F_{IS}. Results for *Mluc*5 was not included because it was monomorphic and not informative. AL = Alice Lake; AS = Armstrong's; CD = Condo; CF = Colony Farm; DI = Deas Island; HL = Hayward Lake; LO = Lillooet; SL = Stave Lake Lodge; SP = Stanley Park; TC = Thompson Creek Farm.

Locus	AL			AS			CD		
	Corrected *P*	F_{IS} WC	F_{IS} RH	Corrected *P*	F_{IS} WC	F_{IS} RH	Corrected *P*	F_{IS} WC	F_{IS} RH
*Mluc*4	0.2502	0.2465	0.2496	1.0000	-0.2786	-0.2822	0.9796	0.0411	0.0421
*Mluc*25	0.7538	-0.0170	-0.0179	0.3939	0.1554	0.0582	0.9796	0.0485	-0.0249
*Mluc*34	0.6400	0.0161	0.0057	0.6178	-0.0164	0.0226	0.9796	-0.0080	0.0116
*Mluc*8	0.2502	0.0192	0.0455	0.6936	0.0333	-0.0016	1.0000	-0.1699	-0.0625
*Mluc*11	0.7538	-0.0226	-0.0482	1.0000	-0.0507	-0.0290	1.0000	-0.0784	-0.0396
*Mluc*29	-	-	-	-	-	-	-	-	-
*Mluc*1	0.7538	-0.0686	-0.0609	0.2752	0.3095	0.1458	0.9796	-0.0739	-0.0966
*Mluc*7	0.2502	0.1004	0.0726	0.6936	-0.0122	-0.0100	0.9796	-0.0472	-0.0360
*Mluc*30	0.2502	0.0653	0.0479	**0.0400**	0.1686	0.1177	0.9796	0.0742	0.0319

Locus	CF			DI			HL		
	Corrected *P*	F_{IS} WC	F_{IS} RH	Corrected *P*	F_{IS} WC	F_{IS} RH	Corrected *P*	F_{IS} WC	F_{IS} RH
*Mluc*4	1.0000	0.0486	0.0283	0.2910	0.3010	0.3070	0.7533	0.0717	0.0734
*Mluc*25	1.0000	-0.0123	-0.0219	0.8354	0.0229	0.0033	0.6216	0.0454	0.0626
*Mluc*34	1.0000	-0.0667	-0.0509	0.8806	-0.0978	-0.0303	0.7533	0.0295	0.0199
*Mluc*8	1.0000	-0.0916	-0.0376	0.8806	-0.0881	-0.0250	0.7533	-0.0366	-0.0138
*Mluc*11	1.0000	-0.1932	-0.0829	1.0000	-0.1915	-0.1239	0.8022	-0.0761	-0.0530
*Mluc*29	1.0000	-0.0073	-0.0074	1.0000	-0.0084	-0.0003	1.0000	-0.0115	-0.0005
*Mluc*1	1.0000	-0.0800	-0.0649	0.1472	0.1022	0.2295	0.3879	0.1396	0.2722
*Mluc*7	1.0000	0.0007	-0.0069	0.8354	0.0189	-0.0010	0.7533	0.0288	-0.0019
*Mluc*30	1.0000	0.0417	0.0177	**0.0405**	0.1594	0.1310	0.2070	0.1126	0.1023

Locus	LO			SL			SP		
	Corrected *P*	F_{IS} WC	F_{IS} RH	Corrected *P*	F_{IS} WC	F_{IS} RH	Corrected *P*	F_{IS} WC	F_{IS} RH
*Mluc*4	**0.0184**	0.6130	0.6326	1.0000	-0.0275	-0.0283	1.0000	-0.1892	-0.1926
*Mluc*25	1.0000	0.0291	-0.0480	1.0000	-0.0024	-0.0260	1.0000	0.0490	0.0023
*Mluc*34	0.8021	0.0732	0.0246	1.0000	0.0261	-0.0031	1.0000	-0.0501	-0.0625
*Mluc*8	1.0000	-0.3027	-0.0933	0.8072	0.0000	0.0396	1.0000	-0.0104	-0.0268
*Mluc*11	1.0000	-0.1933	-0.1014	1.0000	-0.1834	-0.0818	1.0000	-0.2022	-0.2061
*Mluc*29	-	-	-	-	-	-	-	-	-
*Mluc*1	1.0000	-0.1351	-0.1378	1.0000	-0.1674	-0.0686	1.0000	-0.1378	-0.1131
*Mluc*7	1.0000	-0.1507	-0.1127	1.0000	-0.0604	-0.0642	1.0000	-0.0159	-0.0301
*Mluc*30	0.2936	0.0838	0.0522	**0.0312**	0.1863	0.1505	0.1408	0.1434	0.1172

Locus	TC		
	Corrected *P*	F_{IS} WC	F_{IS} RH
*Mluc*4	1.0000	0.0552	0.0565
*Mluc*25	0.7223	0.1262	0.0783
*Mluc*34	1.0000	0.0199	-0.0114

*Mluc*8	1.0000	-0.1500	-0.0625
*Mluc*11	1.0000	-0.0973	-0.0649
*Mluc*29	1.0000	-0.0233	-0.0238
*Mluc*1	1.0000	-0.1692	-0.1020
*Mluc*7	0.9639	0.0146	0.0214
*Mluc*30	0.0702	0.1567	0.1268

Table S4. Tests of heterozygote excess in each geographic population using Genepop program (Version 4.8.3). P-values were corrected for multiple comparisons within each geographic population using the `p.adjust` function with Benjamini and Hochberg method in `stats` R package. WC was the Weir and Cockerham's estimator of F_{IS}. RH was the Robertson and Hill's estimator of F_{IS}. Results for *Mluc*5 was not included because it was monomorphic and not informative. AL = Alice Lake; AS = Armstrong's; CD = Condo; CF = Colony Farm; DI = Deas Island; HL = Hayward Lake; LO = Lillooet; SL = Stave Lake Lodge; SP = Stanley Park; TC = Thompson Creek Farm.

**Locus**	**AL**			**AS**			**CD**		
	**Corrected**	F_{IS}		**Corrected**	F_{IS}		**Corrected**	F_{IS}	
	P	**WC**	**RH**	***P***	**WC**	**RH**	***P***	**WC**	**RH**
*Mluc*4	0.9825	0.2465	0.2496	0.995	-0.2786	-0.2822	0.8417	0.0411	0.0421
*Mluc*25	0.9825	-0.0170	-0.0179	0.995	0.1554	0.0582	0.8417	0.0485	-0.0249
*Mluc*34	0.9825	0.0161	0.0057	0.995	-0.0164	0.0226	0.8417	-0.0080	0.0116
*Mluc*8	0.9825	0.0192	0.0455	0.995	0.0333	-0.0016	0.6520	-0.1699	-0.0625
*Mluc*11	0.9825	-0.0226	-0.0482	0.995	-0.0507	-0.0290	0.8417	-0.0784	-0.0396
*Mluc*29	-	-	-	-	-	-	-	-	-
*Mluc*1	0.9825	-0.0686	-0.0609	0.995	0.3095	0.1458	0.8417	-0.0739	-0.0966
*Mluc*7	0.9825	0.1004	0.0726	0.995	-0.0122	-0.0100	0.8417	-0.0472	-0.0360
*Mluc*30	0.9825	0.0653	0.0479	0.995	0.1686	0.1177	0.8417	0.0742	0.0319

**Locus**	**CF**			**DI**			**HL**		
	**Corrected**	F_{IS}		**Corrected**	F_{IS}		**Corrected**	F_{IS}	
	P	**WC**	**RH**	***P***	**WC**	**RH**	***P***	**WC**	**RH**
*Mluc*4	0.8924	0.0486	0.0283	0.9963	0.3010	0.3070	0.9925	0.0717	0.0734
*Mluc*25	0.5240	-0.0123	-0.0219	0.9963	0.0229	0.0033	0.9925	0.0454	0.0626
*Mluc*34	0.2010	-0.0667	-0.0509	0.9915	-0.0978	-0.0303	0.9925	0.0295	0.0199
*Mluc*8	0.2010	-0.0916	-0.0376	0.9915	-0.0881	-0.0250	0.9925	-0.0366	-0.0138
*Mluc*11	0.2010	-0.1932	-0.0829	0.9915	-0.1915	-0.1239	0.9925	-0.0761	-0.0530
*Mluc*29	0.9930	-0.0073	-0.0074	0.9963	-0.0084	-0.0003	0.9925	-0.0115	-0.0005
*Mluc*1	0.5240	-0.0800	-0.0649	0.9963	0.1022	0.2295	0.9925	0.1396	0.2722
*Mluc*7	0.7137	0.0007	-0.0069	0.9963	0.0189	-0.0010	0.9925	0.0288	-0.0019
*Mluc*30	0.8924	0.0417	0.0177	0.9963	0.1594	0.1310	0.9925	0.1126	0.1023

**Locus**	**LO**			**SL**			**SP**		
	**Corrected**	F_{IS}		**Corrected**	F_{IS}		**Corrected**	F_{IS}	
	P	**WC**	**RH**	***P***	**WC**	**RH**	***P***	**WC**	**RH**
*Mluc*4	0.9999	0.6130	0.6326	0.9296	-0.0275	-0.0283	0.6525	-0.1892	-0.1926
*Mluc*25	0.9607	0.0291	-0.0480	0.8384	-0.0024	-0.0260	0.6934	0.0490	0.0023
*Mluc*34	0.9607	0.0732	0.0246	0.9296	0.0261	-0.0031	0.6525	-0.0501	-0.0625
*Mluc*8	**0.0304**	-0.3027	-0.0933	0.9296	0.0000	0.0396	0.6525	-0.0104	-0.0268
*Mluc*11	0.9099	-0.1933	-0.1014	0.8384	-0.1834	-0.0818	0.6525	-0.2022	-0.2061
*Mluc*29	-	-	-	-	-	-	-	-	-
*Mluc*1	0.9607	-0.1351	-0.1378	0.8384	-0.1674	-0.0686	0.6525	-0.1378	-0.1131
*Mluc*7	0.3648	-0.1507	-0.1127	0.8384	-0.0604	-0.0642	0.6525	-0.0159	-0.0301
*Mluc*30	0.9999	0.0838	0.0522	0.9969	0.1863	0.1505	0.9930	0.1434	0.1172

**Locus**	**TC**		
	**Corrected**	F_{IS}	
	P	**WC**	**RH**
*Mluc*4	0.9931	0.0552	0.0565
*Mluc*25	0.9931	0.1262	0.0783
*Mluc*34	0.9931	0.0199	-0.0114

*Mluc*8	0.2439	-0.1500	-0.0625
*Mluc*11	0.9931	-0.0973	-0.0649
*Mluc*29	0.9931	-0.0233	-0.0238
*Mluc*1	0.9931	-0.1692	-0.1020
*Mluc*7	0.9931	0.0146	0.0214
*Mluc*30	0.9931	0.1567	0.1268

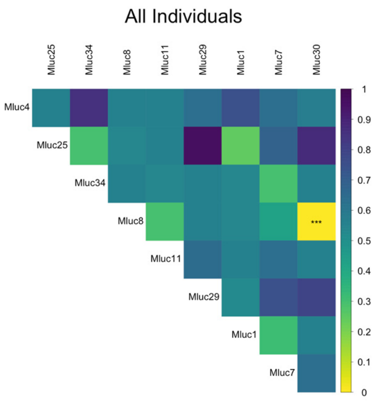
All Individuals
Mluc25
Mluc34
Mluc8
Mluc11
Mluc29
Mluc1
Mluc7
Mluc30
Mluc4
***
1
0.9
0.8
0.7
0.6
0.5
0.4
0.3
0.2
0.1
0

(a)

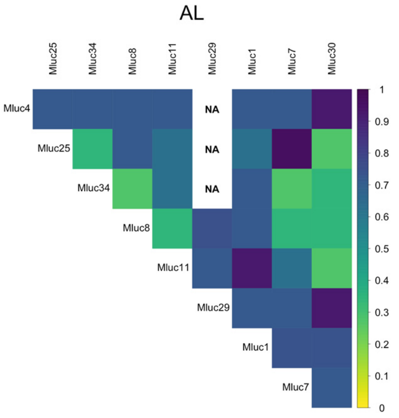
AL
Mluc25
Mluc34
Mluc8
Mluc11
Mluc29
Mluc1
Mluc7
Mluc30
Mluc4
NA
1
0.9
0.8
0.7
0.6
0.5
0.4
0.3
0.2
0.1
0

(b)

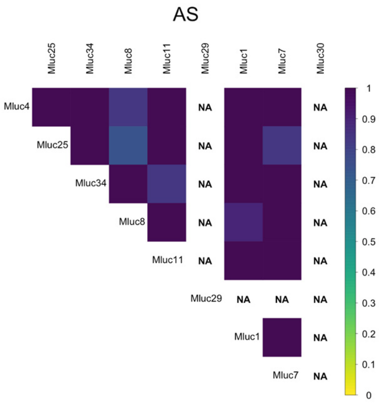
AS
Mluc25
Mluc34
Mluc8
Mluc11
Mluc29
Mluc1
Mluc7
Mluc30
Mluc4
NA
1
0.9
0.8
0.7
0.6
0.5
0.4
0.3
0.2
0.1
0

(c)

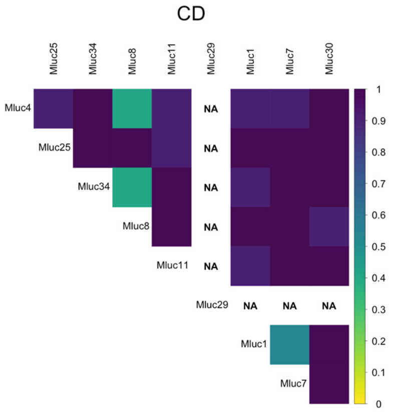
CD
Mluc25
Mluc34
Mluc8
Mluc11
Mluc29
Mluc1
Mluc7
Mluc30
Mluc4
NA
1
0.9
0.8
0.7
0.6
0.5
0.4
0.3
0.2
0.1
0

(d)

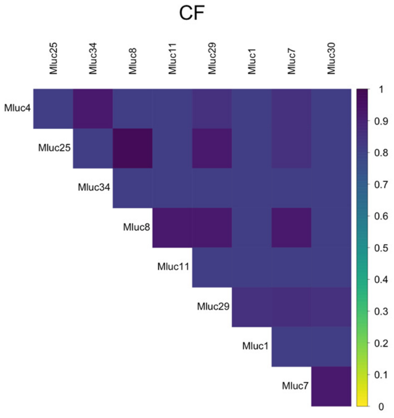
CF
Mluc25
Mluc34
Mluc8
Mluc11
Mluc29
Mluc1
Mluc7
Mluc30
Mluc4
1
0.9
0.8
0.7
0.6
0.5
0.4
0.3
0.2
0.1
0

(e)

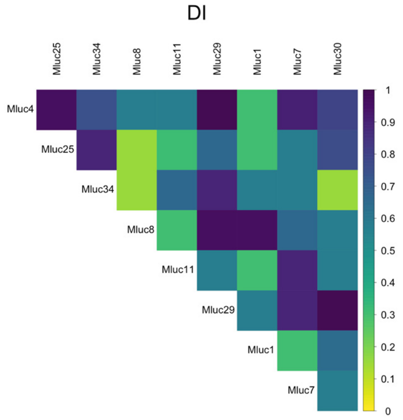
DI
Mluc25
Mluc34
Mluc8
Mluc11
Mluc29
Mluc1
Mluc7
Mluc30
Mluc4
1
0.9
0.8
0.7
0.6
0.5
0.4
0.3
0.2
0.1
0

(f)

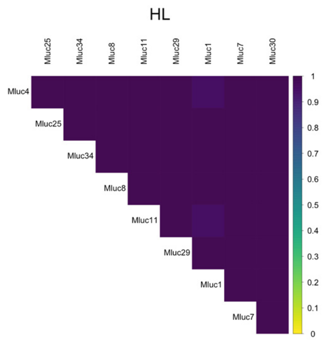

(g)

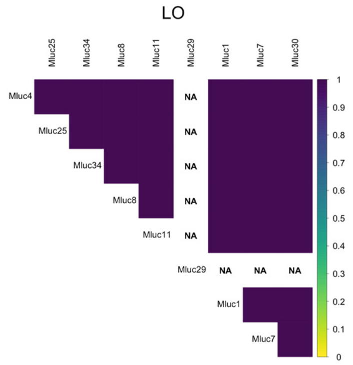

(h)

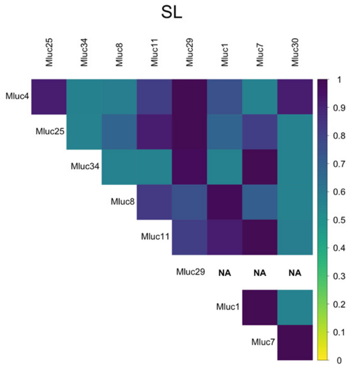

(i)

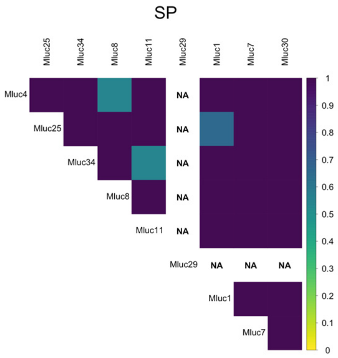

(j)

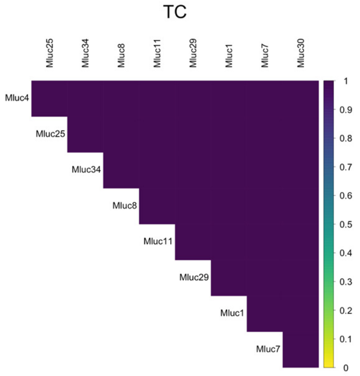

(k)

Figure S3. Linkage disequilibrium tested for each pair of loci in all individuals and in each geographic population using Genepop program (Version 4.8.3). Colors were p-values adjusted for multiple comparisons within all individuals and each geographic population using the `p.adjust` function with Benjamini and Hochberg method in `stats` R package. Significant p-values at 0.05, 0.01, 0.001 were labeled with *, **, *** respectively. Results for *Mluc*5 was not included because it was monomorphic and not informative. Linkage disequilibrium was tested in: (**a**) All individuals;

(**b**) Alice Lake (AL); (**c**) Armstrong's (AS); (**d**) Condo (CD); (**e**) Colony Farm (CF); (**f**) Deas Island (DI); (**g**) Hayward Lake (HL); (**h**) Lillooet (LO); (**i**) Stave Lake Lodge (SL); (**j**) Stanley Park (SP); (**k**) Thompson Creek Farm (TC).

Table S5. Pairwise geographic distance matrix calculated as great circle distance using the `pointDistance` function in `raster` R package (Version 3.6.26), with "longlat" set to TRUE. Distances were in kilometer; AL = Alice Lake; AS = Armstrong's; CD = Condo; CF = Colony Farm; DI = Deas Island; HL = Hayward Lake; LO = Lillooet; (**i**) SL = Stave Lake Lodge; SP = Stanley Park; TC = Thompson Creek Farm.

	AL	AS	CD	CF	DI	HL	LO	SL	SP
AS	466.999								
CD	474.831	16.285							
CF	65.76	444.33	450.185						
DI	73.688	463.465	468.964	21.745					
HL	83.368	411.721	417.493	32.74	52.211				
LO	128.207	396.279	407.39	167.544	186.042	157.971			
SL	81.46	412.076	417.927	32.262	52.213	2.169	156.024		
SP	54.444	468.035	474.165	25.256	20.03	57.453	172.292	56.695	
TC	83.554	415.919	421.571	29.092	47.683	5.278	162.284	6.589	54.191

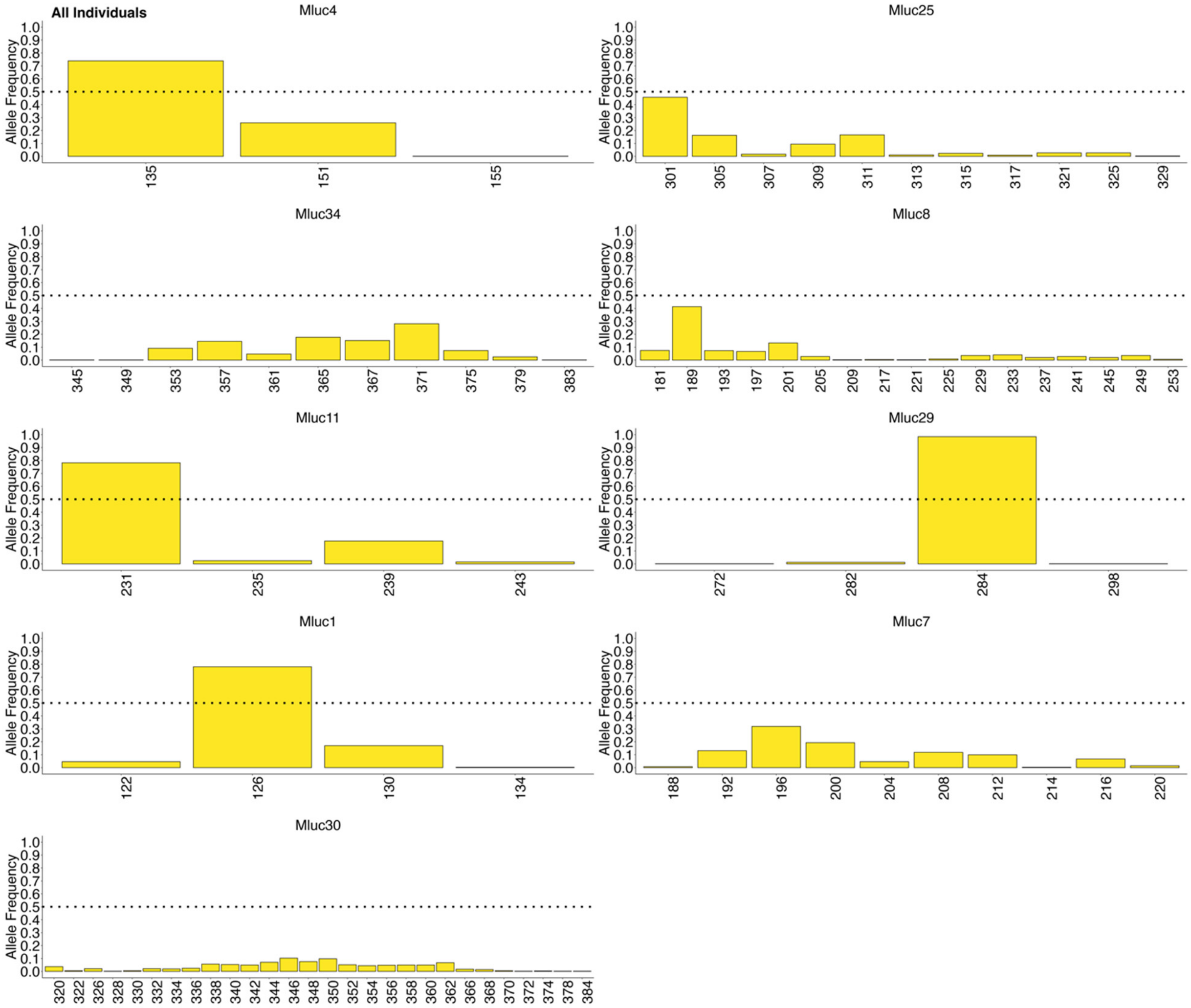

Figure S4. Allele frequencies of the 9 polymorphic microsatellite loci in all individuals. A dotted line is drawn at allele frequency of 0.5.

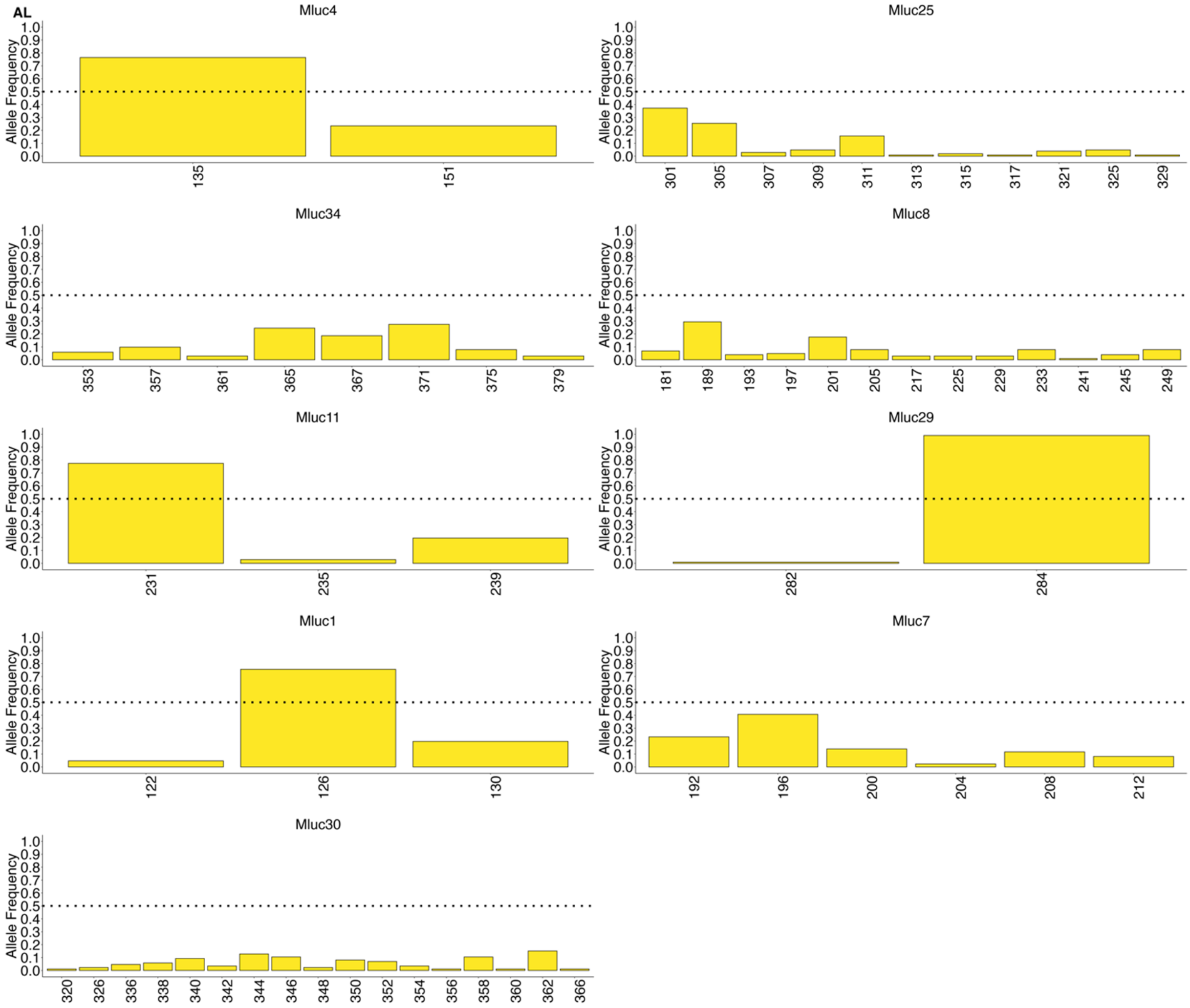

Figure S5. Allele frequencies of the 9 polymorphic microsatellite loci in Alice Lake (AL) population. A dotted line is drawn at allele frequency of 0.5.

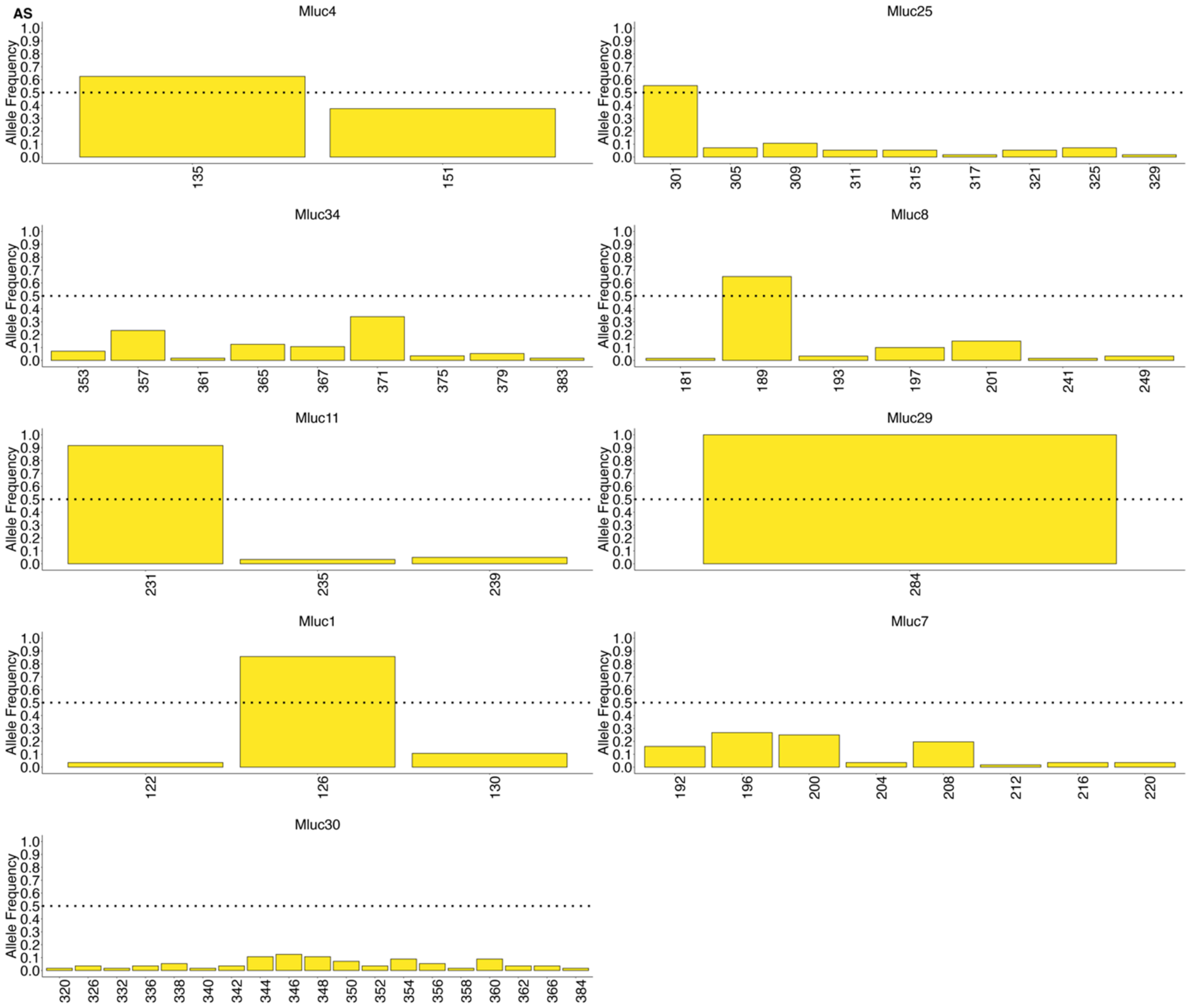

Figure S6. Allele frequencies of the 9 polymorphic microsatellite loci in Armstrong's (AS) population. A dotted line is drawn at allele frequency of 0.5.

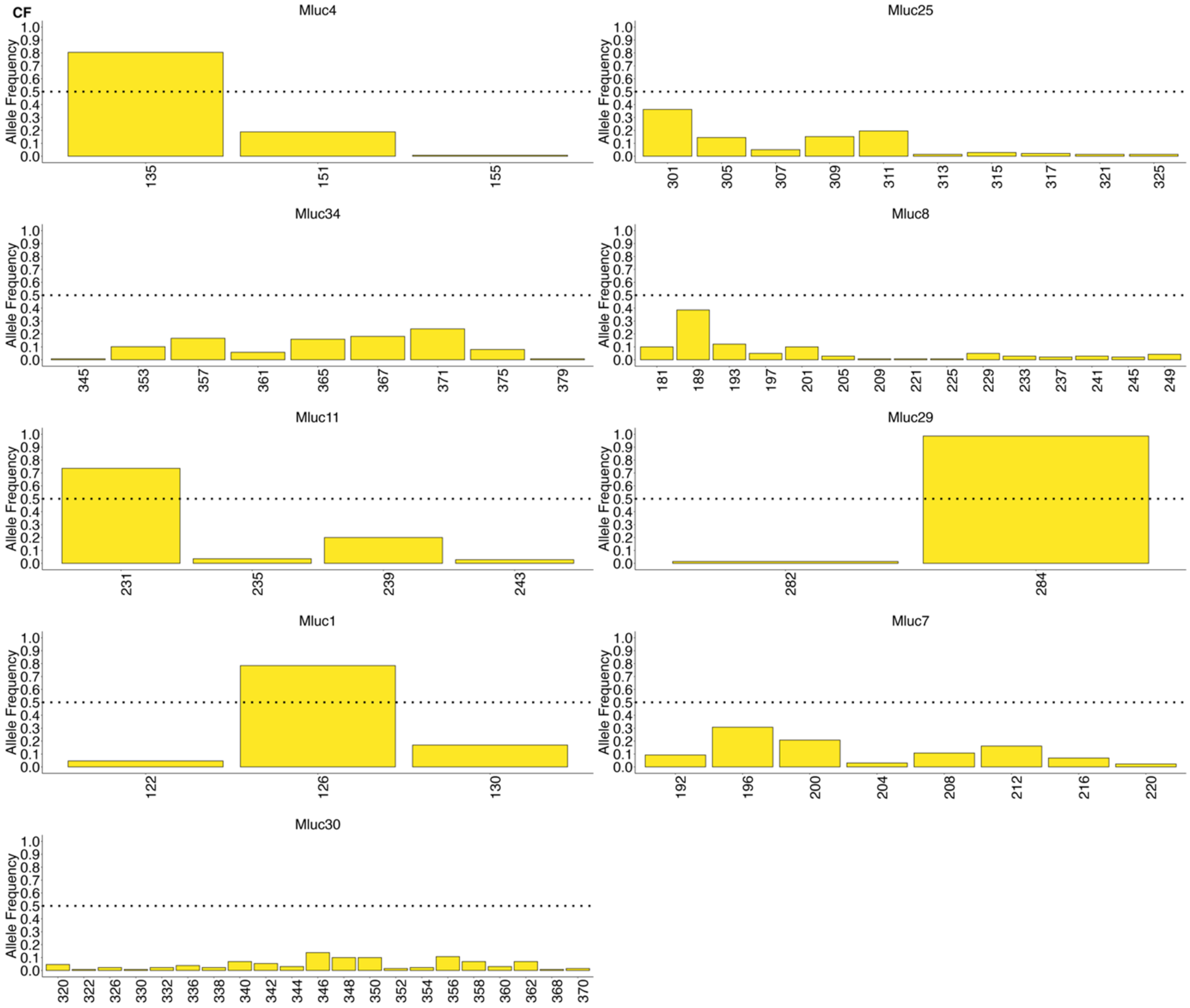

Figure S7. Allele frequencies of the 9 polymorphic microsatellite loci in Colony Farm (CF) population. A dotted line is drawn at allele frequency of 0.5.

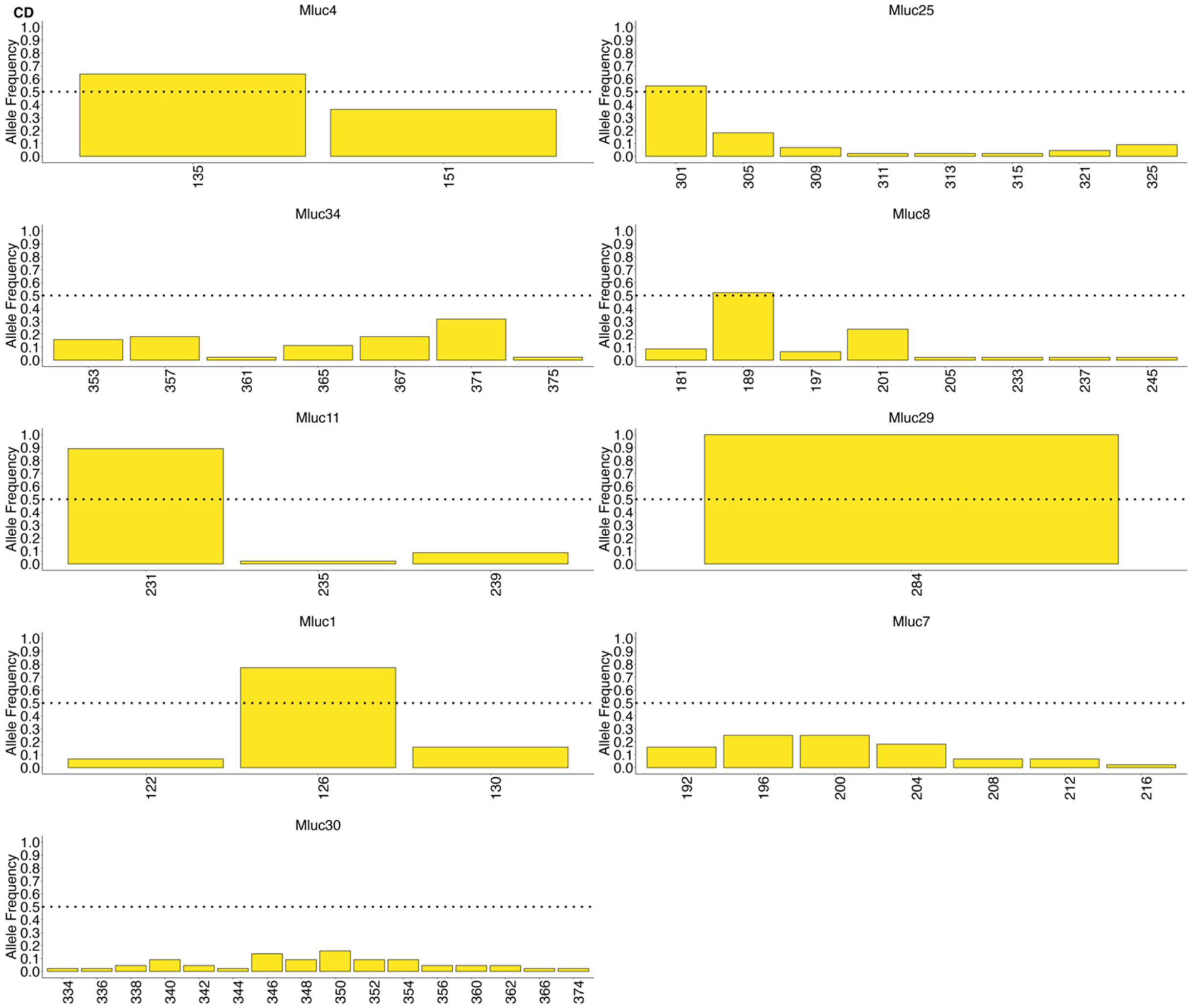

Figure S8. Allele frequencies of the 9 polymorphic microsatellite loci in Condo (CD) population. A dotted line is drawn at allele frequency of 0.5.

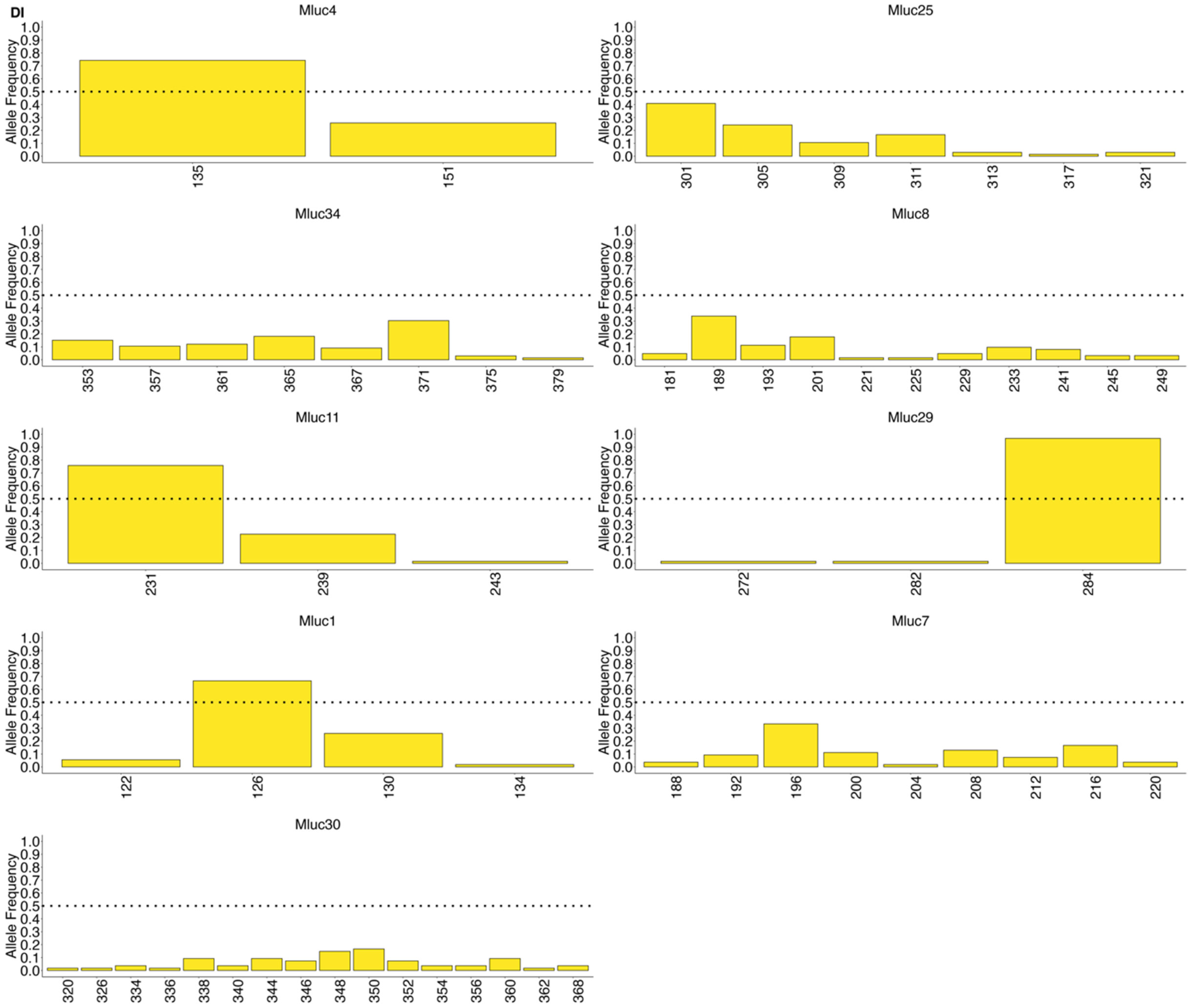

Figure S9. Allele frequencies of the 9 polymorphic microsatellite loci in Deas Island (DI) population. A dotted line is drawn at allele frequency of 0.5.

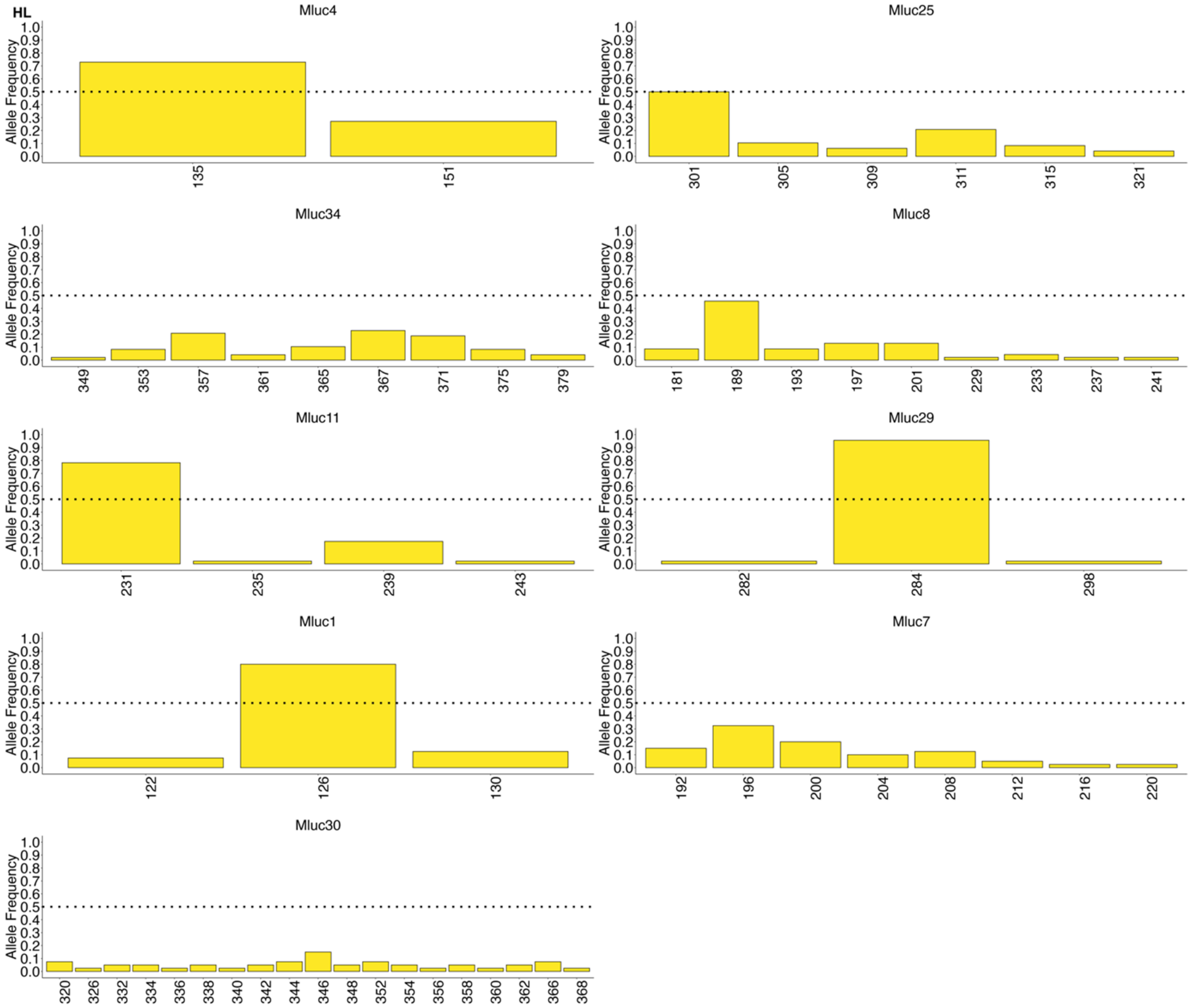

Figure S10. Allele frequencies of the 9 polymorphic microsatellite loci in Hayward Lake (HL) population. A dotted line is drawn at allele frequency of 0.5.

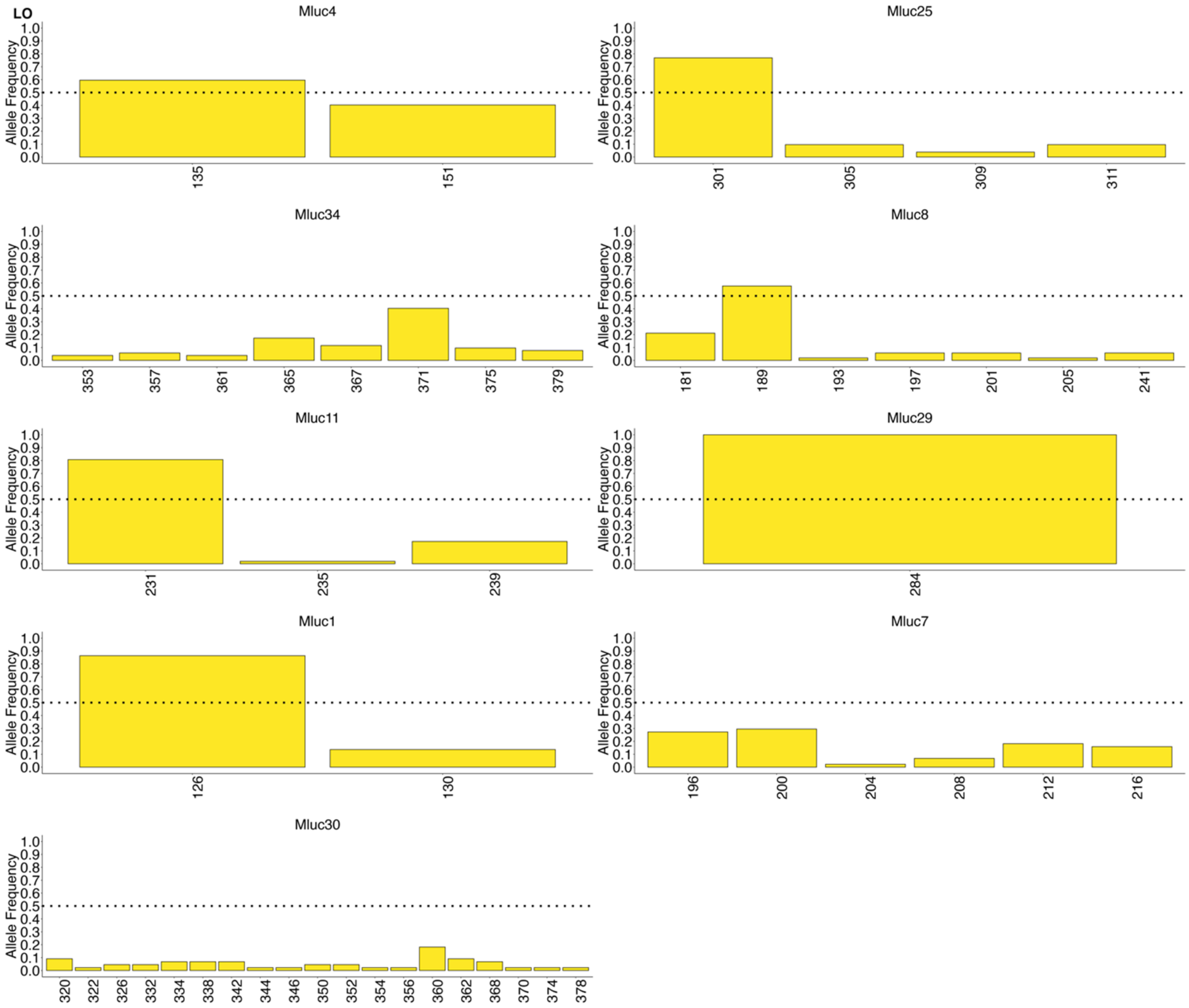

Figure S11. Allele frequencies of the 9 polymorphic microsatellite loci in Lillooet (LO) population. A dotted line is drawn at allele frequency of 0.5.

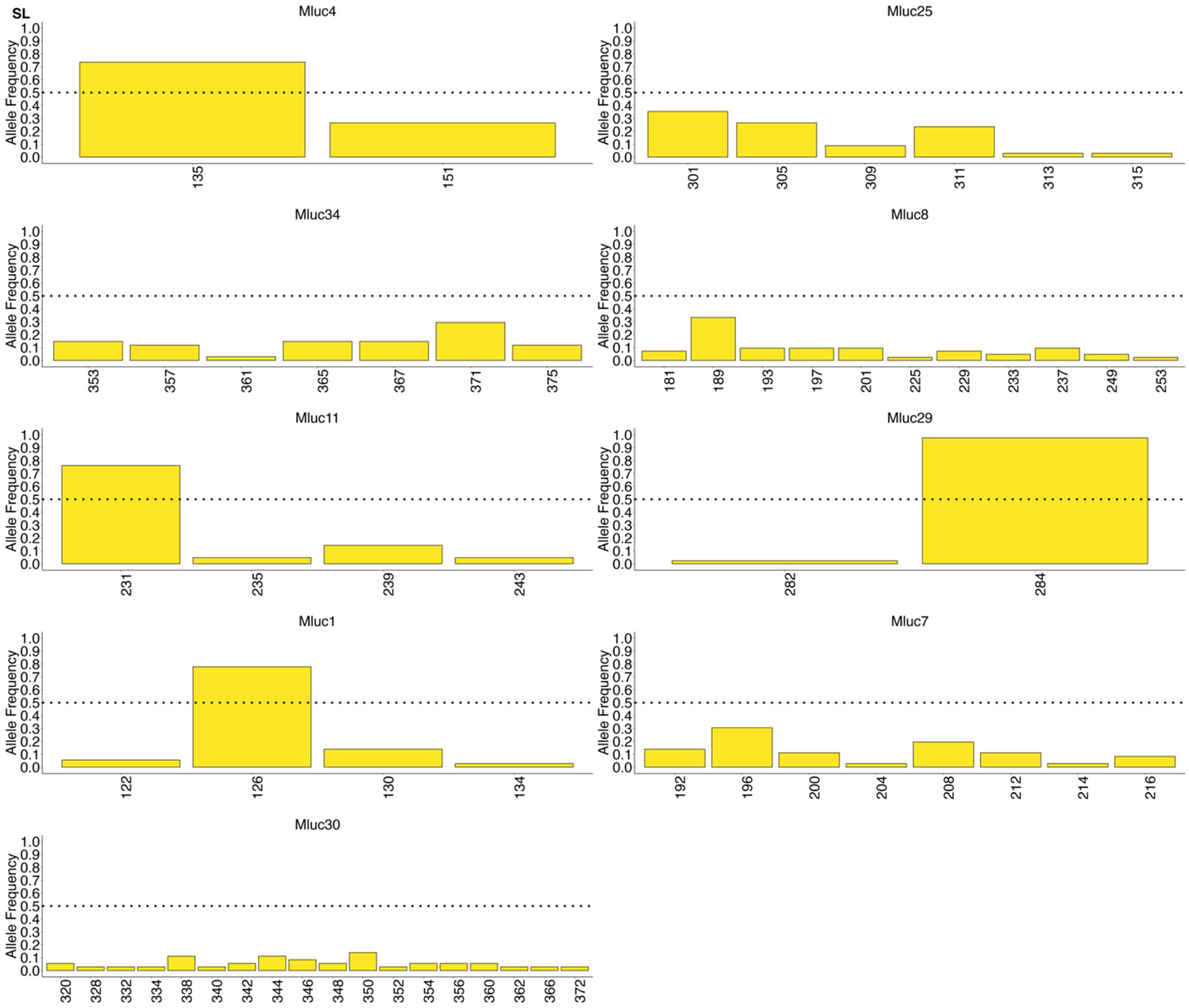

Figure S12. Allele frequencies of the 9 polymorphic microsatellite loci in Stave Lake Lodge (SL) population. A dotted line is drawn at allele frequency of 0.5.

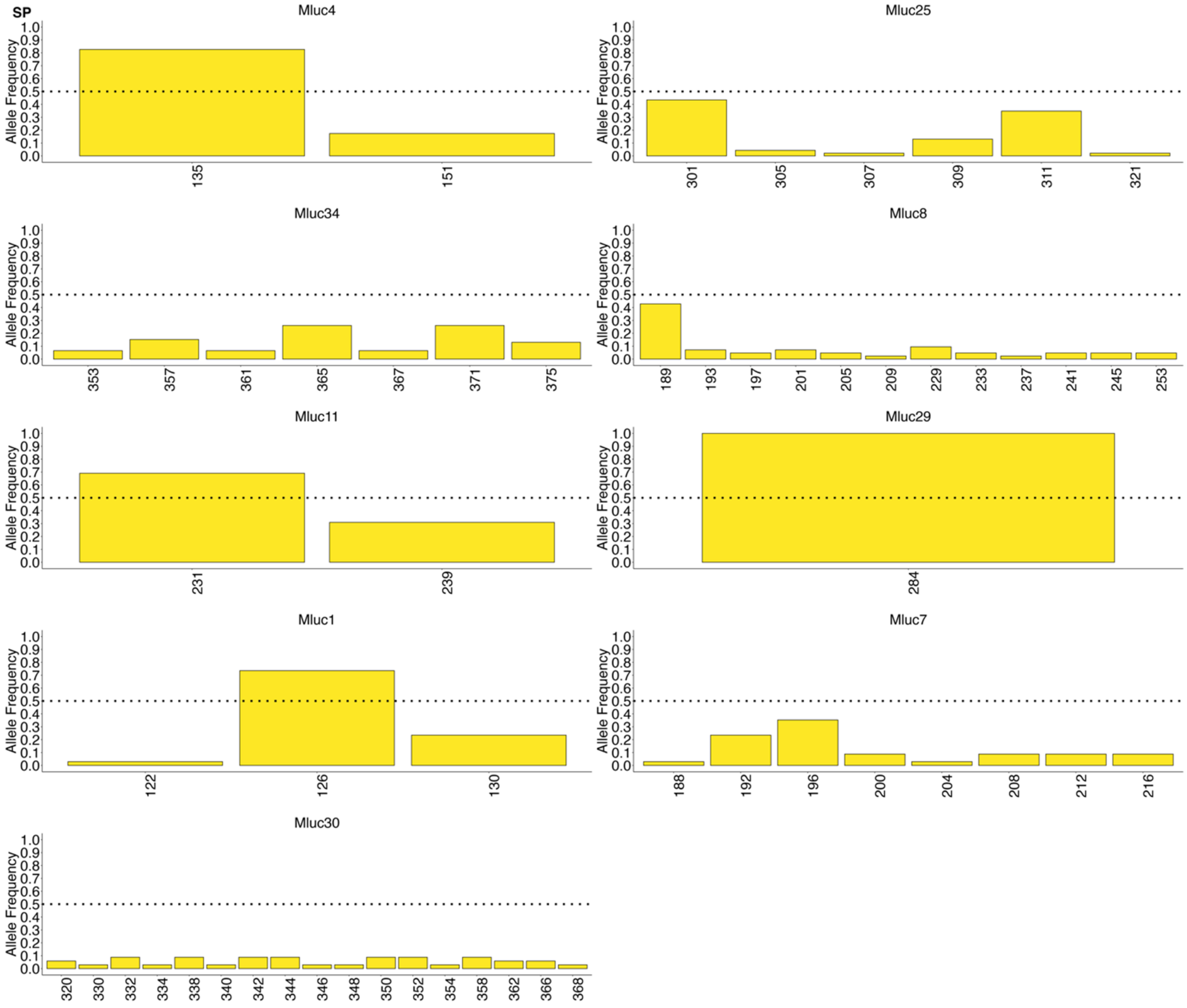

Figure S13. Allele frequencies of the 9 polymorphic microsatellite loci in Stanley Park (SP) population. A dotted line is drawn at allele frequency of 0.5.

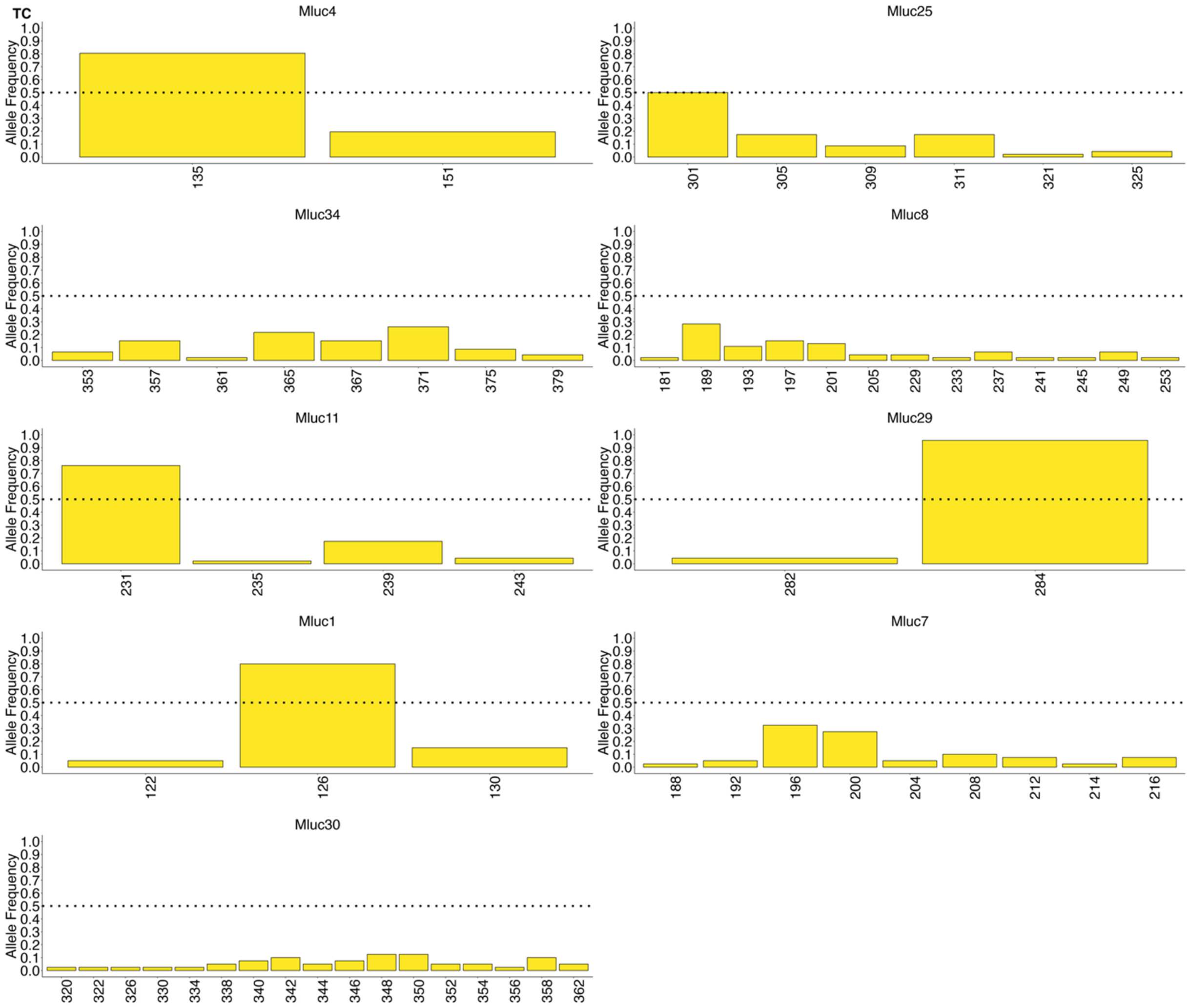

Figure S14. Allele frequencies of the 9 polymorphic microsatellite loci in Thompson Creek Farm (TC) population. A dotted line is drawn at allele frequency of 0.5.
